# Strain-specific and outcome-specific efficacy of probiotics for the treatment of irritable bowel syndrome: A systematic review and meta-analysis

**DOI:** 10.1016/j.eclinm.2021.101154

**Published:** 2021-10-18

**Authors:** Lynne V. McFarland, Tarkan Karakan, Ali Karatas

**Affiliations:** a Department Medicinal Chemistry, School of Pharmacy, University of Washington, 6047 38th Avenue NE, Seattle, WA 98195, United States; b Department of Gastroenterology, Gazi University Faculty of Medicine, Beşevler, Ankara 06500, Turkey

**Keywords:** Probiotic, Irritable bowel syndrome, Gastrointestinal, Meta-analysis, Diarrhea, Constipation

## Abstract

**Background:**

Consistent guidance for choosing an appropriate probiotic for the treatment of irritable bowel syndrome is lacking.

**Methods:**

Literature databases searched included: PubMed, Google Scholar and NIH registry of clinical trials from inception to June 2021. Inclusion criteria: randomized controlled trials (RCTs) enrolling adult or pediatric IBS patients comparing probiotics against controls and ≥ 2 RCTs with common IBS outcome measures within each type of probiotic. Five common measures of IBS symptoms (changes in global Irritable Bowel Syndrome Severity Scoring System or IBS-SSS scores, frequency of global responders, changes in bloating or abdominal pain scores and frequency of abdominal pain relief) were used. This study was registered at Prospero (#CRD42018109169).

**Findings:**

We screened 521 studies and included 42 randomized controlled trials (45 treatment arms, *N* = 3856). Four probiotics demonstrated significant reduction in abdominal pain relief: *B. coagulans* MTCC5260 (RR= 4^.^9, 95% C.I. 3^.^3, 7^.^3), *L. plantarum* 299v (RR= 4^.^6, 95% CI 1^.^9, 11^.^0), *S. boulardii* CNCM I-745 (RR= 1^.^5, 95% C.I. 1^.^1, 2^.^1) and *S. cerevisiae* CNCM I-3856 (RR= 1^.^3, 95% C.I. 1^.^04, 1^.^6). Mild-moderate adverse events were reported in 51% of the trials, none were more associated with the probiotic compared to controls.

**Interpretation:**

Although the analysis of probiotic efficacy was limited by the diversity of IBS outcomes used in trials and lack of confirmatory trials for some strains, six single-strain probiotics and three different types of probiotic mixtures showed significant efficacy for at least one IBS outcome measure. These results might be relevant to clinical practice and policy.

**Research in context**

**Evidence before this study**

Guidance for choosing an appropriate probiotic for the treatment of irritable bowel syndrome is lacking and evidence from reviews and meta-analyses are conflicting. To assess the efficacy and safety of probiotics for the treatment of irritable bowel syndrome (IBS) within specific strain sub-groups, inclusion criteria required ≥2 trials with common IBS outcome measures within each type of probiotic.

**Added value of this study**

Fourteen different probiotic types were analyzed (9 single strains and 5 multi-strain mixtures). Nine probiotic types had at least one outcome with significant efficacy, four probiotics showed no efficacy in any of the outcomes. Four probiotics demonstrated significant reduction in abdominal pain relief: *B. coagulans* MTCC5260, *L. plantarum* 299v, *S. boulardii* CNCMI-745 and *S. cerevisiae* CNCM I-3856.

**Implications of all the available evidence**

Although the analysis of probiotics was limited by the diversity of IBS outcomes used in trials, the study identified nine specific probiotics that are effective for IBS patients and might inform clinical practice.

Alt-text: Unlabelled box

## 1 Introduction

Irritable bowel syndrome (IBS) is now included in the spectrum of disorders of gut-brain interactions and is characterized by recurrent abdominal pain, bloating and changes in bowel habits, which may include diarrhea or constipation. The prevalence of IBS varies by geographic region but has been reported in 1–18% of the general population [1,2]. Symptoms are used to classify sub-types of IBS: diarrhea-predominant IBS (IBS-D), constipation-predominant IBS (IBS-C) or mixed/alternating symptoms (IBS-M). Major risk factors for IBS include female gender, family history of IBS, and environmental triggers such as changes in diet or lifestyle or stress [2]. The pathophysiology of IBS involves chronic mucosal inflammation, alterations in intestinal epithelial and immune functions, visceral hypersensitivity, increased intestinal permeability and dysbiosis of the intestinal microflora. Treatment guidelines recommend medications focused on IBS symptoms, diets low in fermentable types of oligosaccharides, rifaximin and the use of probiotics [3,4]. Choosing an appropriate probiotic for patients can be challenging due to the diversity of different types of available probiotics, efficacy is strain-specific and the paucity of randomized clinical trials for some probiotic strains [5,6]. Recommendations from the European Society for Pediatric Gastroenterology, Hepatology and Nutrition (ESPGHAN) and the American Gastroenterology Association (AGA) suggest efficacy be based only on probiotics with at least two randomized, controlled trials (RCTs) and efficacy should be determined for sub-groups of identical probiotic strains [7,8]. Unfortunately, many meta-analyses have not followed these recommendations and have not been able to determine which specific probiotic strains might be effective for IBS patients [9,10]. The aim of this study is to determine which probiotic strains are safe and effective for the treatment of IBS, accounting for both strain-specificity and based on probiotics with at least one confirmatory trial.

## 2 Methods

### 2.1 Search strategy and study selection

This study was designed and reported according to the Preferred Reporting Items for Systematic Reviews and Meta-analysis (PRISMA) statement [11]. The initial protocol for this study is available in the Supplementary appendix (Text 1) and described below with slight revisions. This review is an update from a prior meta-analysis of probiotics for IBS, but includes trials and recommendations published in the subsequent 13 years [12]. PubMed, Google Scholar and NIH registry of clinical trials were searched from database inception to June 2021, unrestricted by language or year of publication. Non-English papers were translated by one author (LM) and reviewed. Search strategy was as follows: (“probiotics” [MeSH Terms] OR “probiotics” [All Fields]) AND “irritable bowel syndrome” [MeSH Terms] OR “irritable bowel syndrome” [All Fields] AND “controlled trials”) OR “probiotics” [MESH Terms] AND “IBS-diarrhea” [All Fields] OR “probiotics” (MeSH Terms] AND “IBS-constipation” [All Fields] OR “probiotics” [MeSH Terms] AND “IBS-mixed” [All Fields]). Additional searches were done using known probiotic types. Secondary searches of gray literature included reference lists, authors, reviews, meeting abstracts websites and clinicaltrials.gov for unpublished trials. A recursive search was also performed, using the bibliographies of all obtained articles.

Inclusion criteria included: randomized, controlled clinical trials (RCTs) assessing a well-defined probiotic, adult or pediatric patients diagnosed with IBS and published in peer-reviewed journals. We included only probiotics fulfilling the standard definition (must be living microbe, of adequate dose and having efficacy for a health effect [13]. This definition excludes dead or heat-killed microbes and prebiotics. Each probiotic type (single strain or multi-species mixture) was required to have at least two RCTs per type sharing at least one common IBS outcome. As bacterial and fungal taxonomies shift over time, the most current strain designations are presented in this review and strain identification was confirmed with the original authors or the manufacturer whenever possible.

Exclusion criteria included: non-human studies, early phase 1 or 2 safety or mechanism of action studies, no control group, probiotic not well-described, reviews and duplicate reports. The protocol was registered with Prospero (Prospero #: CRD42018109169). This study did not require ethical approval as based on published studies.

### 2.2 Data extraction

Initial screening of studies and data extraction was done independently by one author (LM), then independently reviewed by one of the other two co-authors (TK or AK) following PRISMA guidelines [11]. Any disagreements were discussed until consensus was reached. The data extracted from each study used a standardized data extraction form for PICOS data: (1) patient population (adult/pediatric, age, country, type of IBS), (2) intervention (type of probiotic or controls used, daily doses, formulation, duration and follow-up times), (3) comparisons (type of control group either placebo or open, unblinded), (4) IBS outcomes reported and (5) study design (randomized, controlled trials, either double blinded or open). For data that were required for these analyses but not reported in the published article, we attempted to contact the author or co-authors to obtain the missing data. Data was extracted from summary estimates from published trials from each individual trial.

### 2.3 Outcomes assessment

As no consensus has been reached for a standardized outcome to evaluate IBS improvements, we included the most common outcomes found in these trials. The primary outcomes that were screened included: (1) “Change in global IBS-Symptom Severity Score [IBS-SSS]”, a continuous outcome comparing the change of overall IBS symptom scores from baseline to end of study; (2) “Frequency of Responders”, a dichotomous outcome defined as the number of patients showing improvement of global IBS symptoms reported by the end of the study (either by physician assessment or by subject interview or diaries); (3) “Change in abdominal pain scores”, a continuous outcome comparing the change of scores for abdominal pain from baseline to end of study; (4) “Frequency reporting abdominal pain relief”, a dichotomous outcome (either by physician assessment or by subject interview or diaries) by the end of the study; (5) “Change in bloating scores”, a continuous outcome comparing the change in scores for bloating from baseline to end of study and (6) “Change in QoL (quality of life) scores”, a continuous outcome comparing the change of scores for quality of life from baseline to end of study. The secondary outcome was the number and types of adverse reactions reported by study participants.

### 2.4 Assessment of study quality and risk of bias

Each included RCT was reviewed and scored independently by at least two of the three co-authors using the Cochrane Collaboration's tool for assessing risk of bias [14]. The risk of bias was graded (high, low or not reported) for each of six domains of bias [selection bias (method of randomization and blinded allocation), performance bias (degree of blinding of study personnel and study subjects), detection bias (outcome assessor blinded), attrition bias (attrition different by group), reporting bias (only *a priori* outcomes reported) and other issues (fraud or miscellaneous)].

### 2.5 Data synthesis and statistical methods

Statistical analysis and generation of forest plots of pooled summary estimates was performed using Stata software version 16 (Stata Corporation, College Station, Texas) with meta-analysis modules [15]. Summary estimates were based on the pooled data from RCTs using the same type (species) of probiotic and sharing a common IBS outcome measure. Dichotomous outcomes were assessed using relative risks (RR) and 95% confidence intervals (C.I.) and continuous outcomes were assessing using standard mean difference (SMD) and 95% C.I. and heterogeneity across trials was evaluated using the I^2^ statistic [15]. Random effects models were used for all meta-analyze and results displayed in forest plots. Publication bias was assessed using funnel plots and the Egger test [16,17]. *A priori* sub-groups included: sub-type of IBS (IBS-C, IBS-D or IBS-M), daily doses of probiotic, type of enrolled subject (adult or pediatric), and study quality.

### 2.6 Role of the funding source

This study was un-funded. All authors had access to the dataset and decided to submit for publication. This study did not require ethical approval.

## 3 Results

### 3.1 Overview of included studies

**Fig. 1:**
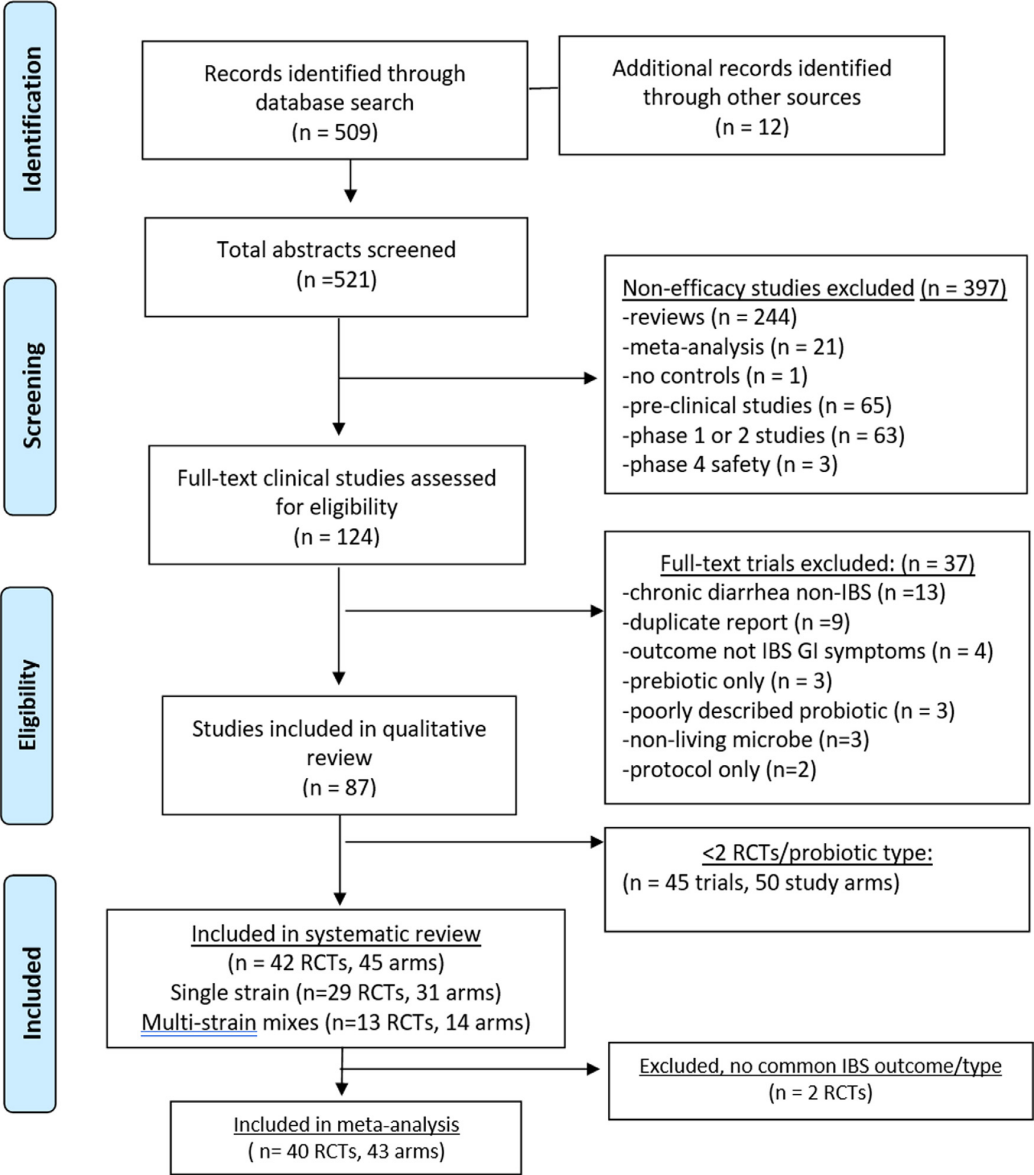
PRISMA study flow-chart of literature search for probiotics for the treatment of irritable bowel syndrome. **Abbreviations**: **GI,** gastrointestinal; **IBS**, irritable bowel syndrome; **RCTs**, randomized controlled trials.

The literature search yielded 521 articles on probiotic use in IBS patients. Reasons for exclusion included non-efficacy studies (*n* = 397) or failure to meet inclusion criteria (*n* = 37), as shown in Fig. 1. Of the 87 RCTs on IBS, 45 RCT (50 treatment arms) were then excluded, as there was only one RCT/type (Supplementary appendix, Table 1). As a result, 42 RCTs (45 treatment arms) were included in the systematic review (*N* = 3856) [18], [19], [20], [21], [22], [23], [24], [25], [26], [27], [28], [29], [30], [31], [32], [33], [34], [35], [36], [37], [38], [39], [40], [41], [42], [43], [44], [45], [46], [47], [48], [49], [50], [51], [52], [53], [54], [55], [56], [57], [58], [59]. For the meta-analysis, two RCTs testing *E. coli* Nissle 1917 were excluded [27,28] as no common IBS outcome measurement was used. Thus, the meta-analysis included a total of 40 RCTs (43 treatment arms). Two trials were translated from the original French language [39,40].

**Table 1:** Efficacy of probiotics by common IBS outcomes in 42 (45 treatment arms) randomized controlled trials .

Probiotic	“Responders” Probiotic No. (%)	“Responders” Controls No. (%)	Less abdominal pain Probiotic No. (%)	Less abdominal pain Control No. (%)	Mean ± SD change in global IBS-SSS scores:Probiotic	Mean ± SD change in global IBS-SSS scores:Control	Mean ± SD change in abdominal pain scores:Probiotic	Mean ± SD change in abdominal pain scores:Control	Reference
*Bac. coagulans* MTCC5260	nd	nd	67/72 (93)^a^	15/69 (22)	−18^.^5 ± 3^.^8^a^	- 8^.^7 ± 4^.^2	- 2^.^9 ± 0^.^8^a^	- 1^.^2 + 0^.^9	Sudha 2018 [18]
*Bac. coagulans* MTCC5260	10/53 (19)^a^	0/55 (0)	45/53 (85)^a^	7/55 (13)	−15^.^8 ± 4^.^0^a^	- 5^.^2 ± 4^.^1	- 4^.^8 ± 2^.^7^a^	- 1^.^6 + 1^.^7	Madempudi 2019 [19]
*Bac. coagulans* MTCC5856	18/26 (69)^a^	1/26 (4)	nd	nd	nd	nd	- 15 ± 28^a^	- 0^.^1 ± 22	Urgesi 2014 [20]
*Bac. coagulans* MTCC5856 + FOS”	nd	nd	nd	nd	nd	nd	- 4^.^2 ± 0^.^3^a^	- 1^.^9 ± 0^.^2	Rogha 2014 [21]
*Bac. coagulans* MTCC5856	nd	nd	nd	nd	nd	nd	- 3^.^9 ± 0^.^3^a^	- 0^.^3 ± 0^.^5	Majeed 2016 [22]
*Bifid. infantis* 35624	nd	nd	nd	nd	nd	nd	- 1^.^0 ± 0^.^2^b^	- 0^.^9 ± 0^.^2^b^	O'Mahony 2005 [23]
*Bifid. infantis* 35624	33/74 (44)	32/76 (42)	32/74 (43)	39/76 (52)	- 0^.^36 ± 0^.^1	- 0^.^42± 0^.^1	- 0^.^4 ± 0^.^1	- 0^.^6 ± 0^.^1	Whorwell 2006 [24] low dose
*Bifid. infantis* 35624	45/72 (62) a	32/76 (42)	42/72 (59)^a^	39/76 (52)	- 0^.^76 + 0^.^1	- 0^.^42± 0^.^1	- 0^.^9 ± 0^.^1	- 0^.^6 ± 0^.^1	Whorwell 2006 [24] medium dose
*Bifid. infantis* 35624	26/71 (37)	32/76 (42)	28/71 (39)	39/76 (52)	- 0^.^38 ± 0^.^1	- 0^.^42± 0^.^1	- 0.7 ± 0^.^1	- 0^.^6 ± 0^.^1	Whorwell 2006 [24] high dose
*Bifid.animalis* DN-173010	88/135 (65) a	63/132 (48)	nd	nd	nd	nd	- 0^.^5 ± 1^.^0^a^	- 0^.^39 ± 0^.^9	Guyonnet 2007 [25]
*Bifid.animalis* DN-173010	nd	nd	nd	nd	- 0^.^4 ± 0^.^3^b^	- 0^.^1 ± 0^.^4^b^	- 0^.^5 ± 1^.^6^b^	- 0^.^1 ± 1^.^2^b^	Agrawal 2009 [26]
*E. coli* Nissle 1917	27/51 (53)	23/48 (48)	33/60 (55)	30/60 (50)	nd	nd	nd	nd	Kruis 2012 [27]
*E. coli* Nissle 1917	nd	nd	nd	nd	- 6^.^7 ± 6^.^8	- 6^.^7 ± 6^.^5	nd	nd	Faghihi 2015 [28]
*L. plantarum* 299v DSN9843	11/25 (44)	7/27 (26)	9/25 (36)	5/27 (18)	nd	nd	- 2^.^0 ± 0^.^1^a^	- 1^.^0 ± 0^.^3	Nobaek 2000 [29]
*L. plantarum* 299v DSN9843	19/20 (95)^a^	3/20 (15)	20/20 (100)^a^	4/20 (20)	nd	nd	nd	nd	Niedezielin 2001 [30]
*L. plantarum* 299v DSN9843	10/29 (35)	11/29 (38)	nd	nd	- 16 ± 82	- 60 ± 45	nd	nd	Simren 2006 [31]
*L. plantarum* 299v DSN9843	82/105 (78)^a^	8/99 (8)	79/98 (81)	8/92 (9)	nd	nd	- 0^.^6 ± nd	- 0^.^3 ± nd	Ducrotte 2012 [32]
*L. plantarum* 299v DSN9843	nd	nd	nd	nd	nd	nd	- 60^.^5 ± 2^.^8	- 54^.^1 ± 6^.^9	Stevenson 2014 [33]
*L. rhamnosus* GG	nd	nd	11/25 (44)	10/25 (40)	nd	nd	- 13 ± 0^.^3	- 1^.^7 ± 0^.^6	Bausserman 2005 [34]
*L. rhamnosus* GG	nd	nd	6/18 (33)^a^	1/19 (5)	nd	nd	- 1^.^5 ± 1^.^5	- 1^.^0 ± 1^.^2	Gawronska 2007 [35]
*L. rhamnosus* GG	nd	nd	34/42 (82)^a^	17/38 (45)	nd	nd	- 1^.^9 ± 1^.^6 a	- 1^.^0 ± 2^.^5	Francavilla 201,0^36^
*L. rhamnosus* GG	nd	nd	nd	nd	- 68 ± 107^a^	- 34 ± 95	nd	nd	Pedersen 2014 [37]
*L. rhamnosus* GG	nd	nd	nd	nd	nd	nd	- 1^.^7 ± 0^.^9 a	- 1^.^2 ± 0^.^8	Kianifar 2015 [38]
*S. boulardii* CNCM I-745	13/16 (81) a	13/18 (72)	6/15 (40)	3/18 (17)	nd	nd	nd	nd	Maupas 1983 [39]
*S. boulardii* CNCM I-745	nd	nd	22/25 (81)^a^	18/30 (56)	nd	nd	nd	nd	Bennani 1990 [40]
*S. boulardii* CNCM I-745	11/45 (25)	9/45 (20)	nd	nd	−0^.^5 ± 0^.^8	- 0^.^5 ± 0^.^8	- 0^.^3 ± 1^.^0	- 0.^.^ ± 1^.^0	Choi 2011 [41]
*S. boulardii* CNCM I-745	nd	nd	nd	nd	nd	nd	−0^.^4 ± 0^.^7	- 0^.^8 ± 0^.^7	Kabir 2011 [42]
*S. boulardii* CNCM I-745	nd	nd	nd	nd	nd	nd	- 0^.^04 ± 0^.^9	- 0^.^3 ± 0^.^5^a^	Abbas 2014 [43]
*S. cerevisiae* I-3856	nd	nd	54/86 (63)^a^	44/93 (47)	nd	nd	−1^.^2 ± 1^.^3	- 0^.^85 ± 1^.^4	Pineton de Chambrun 2015 [44]
*S. cerevisiae* I-3856	nd	nd	57/177 (32)	47/175 (27)	- 3^.^6 ± 4^.^3	−3^.^3 ± 4^.^0	nd	nd	Spiller 2016 [45]
*S. cerevisiae* I-3856	nd	nd	nd	nd	nd	nd	- 1^.^7 ± 0^.^9^a^	- 0^.^4 ± 0^.^7	Gayathri 2020 [46]
3 strain mix	14/37 (38)	10/37 (27)	nd	nd	- 30 ± 80	- 60 ± 90	- 8 ± 30	- 3 ± 30	Simren 2010 [47]
3 strain mix	9/27 (33)	7/25 (28)	nd	nd	- 89 ± 140	- 47 ± 120	- 17 ± 15	- 9 ± 27	Sondergaard 2011 [48]
3 strain mix	35/67 (52)	26/64 (41)	nd	nd	−0^.^1 ± 1^.^0	- 0^.^2 ± 1^.^0	- 0^.^1 ± 1^.^2	- 0^.^6 ± 1^.^2	Begtrup 2013 [49]
4 strain mix	31/41 (76)^a^	17/40 (43)	27/41 (66)^a^	17/40 (43)	- 7^.^7 ± 2^.^0^a^	- 1^.^2 ± 2^.^0	- 4^.^2 ± nd	- 5^.^8± nd	Kajander 2005 [50]
4 strain mix	nd	nd	nd	nd	- 14 ± 10 a^,^^b^	- 3 ± 9^b^	- 3 ± 2.2^a^^,^^b^	0 ± 3^.^0^b^	Kajander 2008 [51]
6 strain mix	17/25 (68) a	9/24 (37)	nd	nd	nd	nd	- 1.2 ± 0.2	- 0.5 ± 0.3	Yoon 2014 [52]
6 strain mix	29/39 (74)	26/42 (62)	nd	nd	nd	nd	- 2 ± 1	- 1.2 ± 1.0	Yoon 2015 [53]
7 strain mix	12/25 (48)^a^	3/25 (12)	nd	nd	- 1^.^0 ± 0^.^2	- 0^.^8 ± 0^.^06	- 0^.^6 ± 0^.^05	- 0^.^3 ± 0^.^1	Ki Cha 2012 [54]
2/4 txt arms: 7 strain mix vs placebo	nd	nd	10/14 (71)^a^	3/12 (25)	- 19. ^.^ ± 5^.^0	- 9^.^8 ± 0^.^5	- 12^.^1 ± 1^.^0	- 7^.^8 ± 7^.^1	Ko 2013 [55]
2/4 txt arms: Herbal txt + 7 strain mix vs herbal control	nd	nd	10/13 (77)	11/14 (78)	- 14^.^5 ± 1^.^3	- 5^.^8 ± 0^.^8	- 10^.^2 ± 0^.^2	- 3^.^0 ± 0^.^6	Ko 2013 [55]
8-strain mix	4/12 (33) a	5/13 (38)	nd	nd	- 35 ± 32	- 7^.^0 ± 31	- 8 ± 12	- 0 ± 8	Kim 2003 [56]
8-strain mix	11/24 (46)	8/24 (33)	nd	nd	nd	nd	nd	nd	Kim 2005 [57]
8-strain mix	44/59 (75)^a^	2/59 (3)	40/59 (68)^a^	0/59 (0)	nd	nd	- 1^.^0 ± 0^.^2^a^	- 0^.^5 ± 0^.^2	Guandalini 2010 [58]
8-strain mix	30/53 (57)^a^	19/51 (37)	nd	nd	- 82 ± 78	- 78 ± 96	nd	nd	Staudaeher 2017 [59]

**Abbreviations**: Bac., Bacillus; Bifid., *Bifidobacterium*; E., Enterococcus; L., *Lactobacillus;* No., number; nd, not done or not reported; P., *Propionibacterium*; S., *Saccharomyces*; SD, standard deviation; Strept., *Streptococcus;***3 strain mix,** “Cultura®”*:* L. *paracasei* 19, L. *acido* La5, *Bifido lactis* Bb12; **4 strain** mix, L. *rhamnosus* GG, L. *rhamnosus* LC705, *Bifido. breve* Bb99, *P. freudenreichii* ssp. *shermanii* JS; **6 strain** mix, “LacClean®” L. *acidophilus* 11906BP, L. *rhamnosus* 12202BP, *Bifido. bifidum* 12199BP, *Bifido. lactis* 11,904 BP, *Bifido. longum* 12,200 BP, *Strept. thermophilus* 11870BP; **7 strain** mix, “DuoLac®:*: Bifido. brevis* 11858BP, *Bifido. lactis* 11903BP, *Bifido. longum* 11860BP*,* L. *acidophilus* 11906BP, L. *rhamnosus* 11868BP, L. plantarum 11867BP, *Strept. thermophilus* 11870BP; **8 strain mix,***Bifido. breve* DSM24732, *Bifido. longum* DSM24736, *Bifido. infantis* DSM24737, L. *acidophilus* DSM24735, L. *plantarum* DSM24730, L. *paracasei* DSM24733, L. *delbruckii* subsp. *bulgaricus* DSM24734, *Strept. thermophilus* DSM24731, originally named VSL#3®, now either Visbiome™ or Vivomixx™ using the De Simone formulation.

a *P* < 0.05 compared to control.

b Estimated standard deviation.

### 3.2 Study population and study design characteristics

The study population were mostly adults (86%), female (66%), with mixed IBS sub-types (60%), diagnosed with Rome II/III criteria (93%), and enrolled in one of 17 countries (Supplementary appendix Table 2). The mean number of participants per RCT was 92/trial. Attrition was generally low (0–5% in 31% of 42 RCTs) or moderate (6–24% in 60% of RCTs).

### 3.3 Probiotic intervention characteristics

The daily dose of the probiotic was typically given at 10^9–^10^10^/day in 81% of RCTs for 4–8 weeks (in 83% of RCTs), as either capsules (57%) or in beverages (16%) and more details are given in Supplementary appendix Table 2. Nearly half (48%) of the 42 RCTs did not include a post-probiotic/control follow-up period, but 21% did follow patients for 2 weeks and 31% of the RCTs had longer follow-up times (3 weeks-1 year).

From the 42 RCTs (total of 45 intervention arms), a total of 14 different types of probiotic products were assessed (Table 1). Sufficient evidence (≥ 2 RCTs) were found for nine different types of single strains of probiotics: *Bacillus (Bac.) coagulans* MTCC5260 (2 RCTs), *Bac. coagulans* MTCC5856 (3 RCTs), *Bifidobacterium (Bif.) infantis* 35624 (2 RCTs, 4 treatment arms), *Bif. animalis* DN-173010 (2 RCTs), *E. coli* Nissle 1917 (2 RCTs), *Lactobacillus* (*L*.) *plantarum* 299v (5 RCTs), *L. rhamnosus* GG (5 RCTs), *Saccharomyces* (*S*.) *boulardii* CNCM I-745 (5 RCTs) and *S. cerevisiae* (I-3856 (3 RCTs). Sufficient evidence (≥ 2 RCTs) was also found for five different types of multi-strain mixtures: a three-strain mixture of *L. paracasei* 19, *L. acidophilus* La5 and *Bif. lactis* Bb12 (3 RCTs), a four-strain mixture of *L. rhamnosus* GG, *L. rhamnosus* LC705, *Bif. breve* Bb99 and *P. shermanii* (2 RCTs), a six-strain mixture of *L. acidophilus* 11906BP, *L. rhamnosus* 12202BP, *Bif. bifidum* 12199BP, *Bif. lactis* 11904, *Bif. longum* 12200, *Streptococcus* (*Strept*.) *thermophilus* 11870BP (2 RCTs), a seven-strain mixture of *Bif. brevis* 11858BP, *Bif. lactis* 11903BP, *Bif. longum* 11860BP, *L. acidophilus* 11868BP*, L. rhamnosus* 11868BP*,* L. *plantarum* 11867BP and *Strept. thermophilus* 11870BP (2 RCTs, 3 treatment arms) and an eight-strain mixture of *Bif. breve* DSM24732, *Bif. longum* DSM24736, *Bif. infantis* DSM24737, *L. acidophilus* DSM24735, *L. plantarum* DSM24730, *L. paracasei* DSM24733, *L. delbruckii* subsp*. bulgaricus* DSM24734 and *Strept. thermophilus* DSM24731 (4 RCTs).

### 3.4 Assessment of study quality

Of the 42 RCTs (and 45 treatment arms) included in the systematic review, six types of bias were evaluated (Supplementary appendix Fig. 1). Most (98%) had an overall low risk of bias. We were unable to perform a sub-group analysis comparing efficacy of specific probiotic strains done in low-risk versus high-risk bias trials, as only one trial had a high risk of overall bias^.46^ Within bias domains, most high risk bias ratings were due to two reasons: 48% did not report if the study group allocator was blinded and 31% did not report the method of randomization used. Of the other domains of study bias (performance, detection, attrition, reporting and other), most were rated of low bias in each of these domains.

### 3.5 Safety

Of the 42 RCTs reviewed, seven (17%) did not report any adverse reaction (AE) data (Supplementary appendix Table 3), while 17 (40%) only included a statement ‘No adverse reactions were noted in the study’, but did not provide further data on frequency by study group or by type of adverse reaction. Three (7%) RCTs only reported total adverse reactions, but not by study group. Only 15 (36%) of the RCTs reported adverse event data by study group (probiotic versus placebo). With the exception of one RCT [21], there were no significant difference in the frequency or types of mild-moderate AEs in probiotic versus control groups. The most common types of reported adverse reactions were mild gastrointestinal complaints (69%) or rash (15%). Infrequent serious AEs associated with probiotic use (vertigo) was reported in one trial [32] and two trials reported serious AEs (hospitalization or not described) in the placebo group [25,27].

### 3.6 Meta-analysis of efficacy for IBS

Among the 40 RCTs included in the meta-analysis, many different IBS outcomes were used, ranging from changes in global IBS symptoms to changes in specific IBS symptoms (abdominal pain, bloating, gas, etc.) to the quality of life indicators or changes in immune system markers. Four of the most commonly reported IBS outcomes are provided in Table 1, which shows the inconsistency of commonly shared IBS outcomes used in these trials, even within the same type of probiotic strain(s). The two most common IBS outcomes assessed improvement in global (overall) IBS symptoms (change in IBS-SSS scores) and the frequency of ‘Responders). Symptom-specific outcomes were commonly reported (change in abdominal pain scores, frequency reporting abdominal pain relief and changes in bloating scores). Other outcomes including IBS quality of life and changes in other IBS symptoms were not consistently reported or used different scoring scales and thus were not used in the meta-analyses. Five IBS outcomes had at least two RCTs with the same outcome and had at least two probiotics of the same strain or mixture sharing that common IBS outcome. Publication bias was difficult to assess by probiotic type, as the number of trials per type was limited. To overcome this limitation, we pooled all probiotic types together for the 20 trials reporting a common outcome (frequency of no abdominal pain by study end) and the funnel plot (Supplementary appendix Fig. 2) showed there was significant publication bias (Egger's test *P* = 0.005). Similar results were found for trials using the other IBS outcomes once we pooled all probiotic types together (data not shown).

**Fig. 2:**
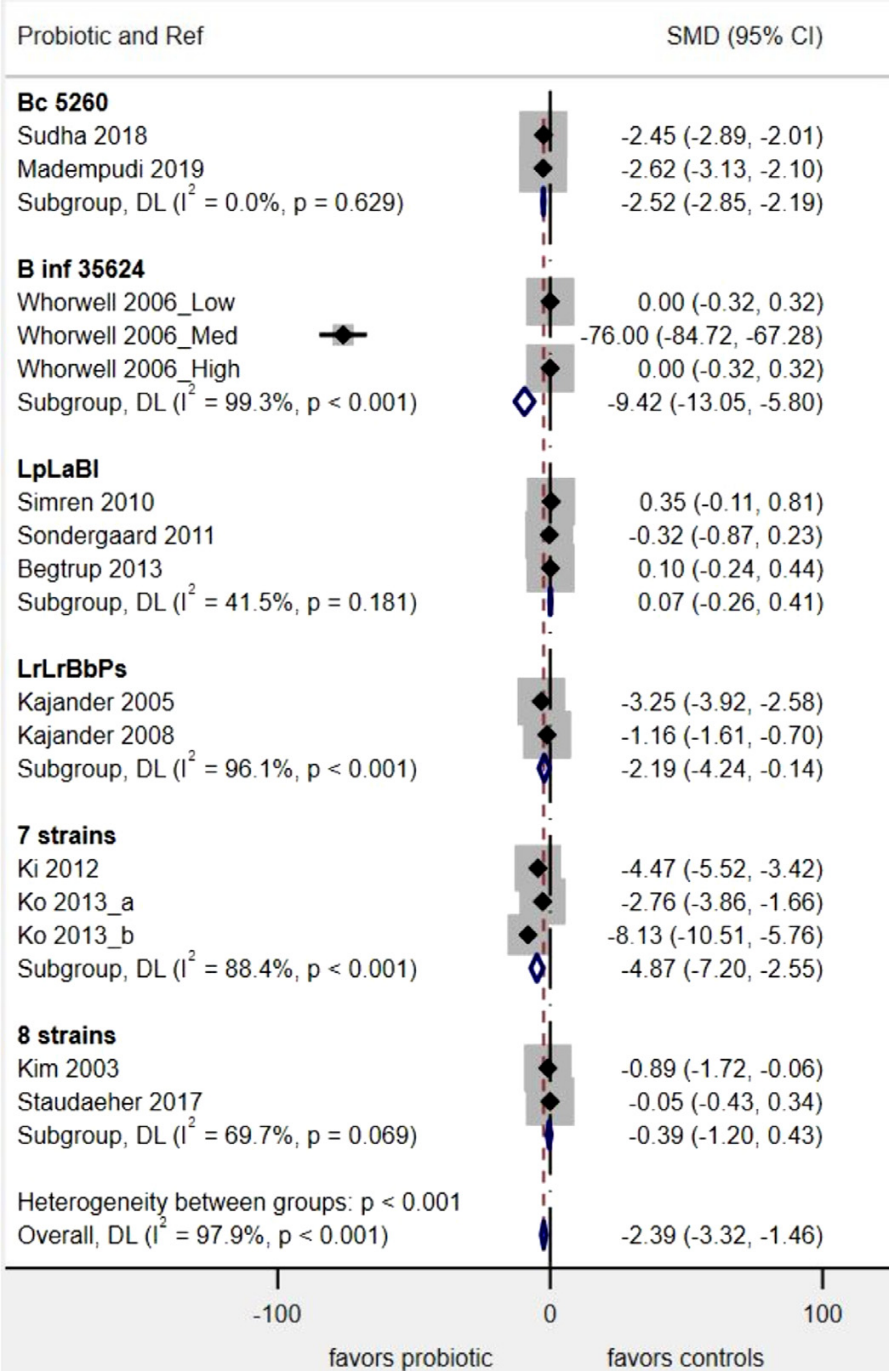
Forest plot of probiotics for the change in IBS symptom scores. Boxes indicate each study's relative risk and horizontal lines indicate each study's 95% confidence intervals, Diamond indicates subgroup's pooled RR and 95% C.I. **Abbreviations**: **Bc**, *Bacillus coagulans*; **B inf**, *Bifidobacterium infantis***;** L., *Lactobacillus;***LpLaBl**, “Cultura®”*:* L. *paracasei* 19, L. *acido* La5, *Bifido lactis* Bb12; **LrLrBbPs,** L. *rhamnosus* GG, L. *rhamnosus* LC705, *Bifido. breve* Bb99, *P. freudenreichii* ssp. *shermanii* JS; **7 strains**, “DuoLac®:*: Bifido. brevis* 11858BP, *Bifido. lactis* 11903BP, *Bifido. longum* 11860BP*,* L. *acidophilus* 11906BP, L. *rhamnosus* 11868BP*,* L. *plantarum* 11867BP, *Strept. thermophilus* 11870BP; **8 strains**, *Bifido. breve* DSM24732, *Bifido. longum* DSM24736, *Bifido. infantis* DSM24737, L. *acidophilus* DSM24735, L. *plantarum* DSM24730, L. *paracasei* DSM24733, L. *delbruckii* subsp. *bulgaricus* DSM24734, *Strept. thermophiles* DSM24731, originally named VSL#3®, now either Visbiome™ or Vivomixx™ using the De Simone formulation.

#### 3.6.1 Change in global IBS-SSS scores

Of the 14 different types of probiotics, six had ≥ 2 RCTs/probiotic type and also reported this shared IBS outcome. Four probiotic types significantly reduced global IBS-SSS scores by the end of the study, as shown in Fig. 2: *Bif. infantis* 35624 (SMD= −9^.^4, 95% C.I. −13^.^0, −5^.^8, *P* < 0.001), a 7-strain mixture (SMD= −4^.^9, 95% C.I. −7^.^2, −2^.^5, *P* < 0.001), *Bac. coagulans* MTCC5260 (SMD= −2^.^5, 95% C.I. −2^.^8, −2^.^2, *P* < 0.001) and a 4-strain mixture (SMD= −2^.^2, 95% C.I. −4^.^2, −0^.^1, *P* = 0.04), while two other types of probiotics (a 3 strain mixture and an 8-strain mixture) showed no significant reduction in IBS-SSS scores. Overall, the heterogeneity in the 21 RCTs with this outcome was high (I^2^= 97.9%), but by stratifying by probiotic type, low heterogeneity (from I^2^=0–41%) was achieved for 2/6 (33%) of the probiotic sub-groups.

#### 3.6.2 Frequency of global responders

Six different types of probiotics had sufficient RCTs/type to be included for this outcome, but none showed a significant increase in the number of responders in global IBS symptoms by the end of the study, as shown in Supplementary appendix Fig. 3. Two probiotics showed a non-statistically significant trend in better responder rates: *L. plantarum* 299v (*P* = 0.06) and a 3-strain mixture (*P* = 0.09). Overall, the heterogeneity in the 16 RCTs with this outcome was high (I^2^= 75%), but by stratifying by probiotic type, low heterogeneity (from I^2^=0–34%) was achieved for 4/6 (67%) of the probiotic sub-groups.

**Fig. 3:**
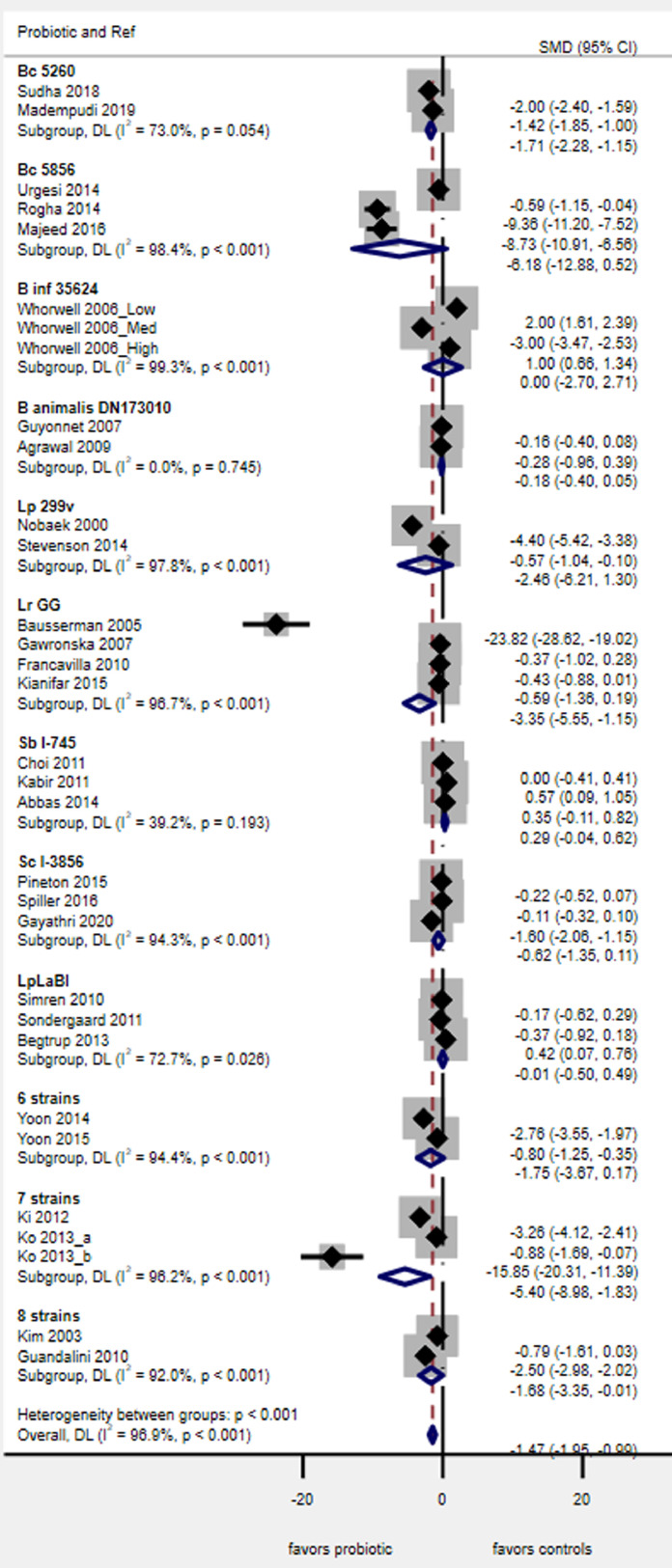
Forest plot of probiotics for the change in IBS abdominal pain scores. Boxes indicate each study's relative risk and horizontal lines indicate each study's 95% confidence intervals, Diamond indicates subgroup's pooled RR and 95% C.I. **Abbreviations: B**, Bifidobacterium; **Bc,***Bacillus coagulans;***Binf**, *Bifidobacterium infantis;***Lp,***Lactobacillus plantarum***Lr,** L. *rhamnosus;***Sb***, Saccharomyces boulardii*; ***Sc***, *Saccharomyces cerevisiae*; **LpLaBl***,* “Cultura®”*:* L. *paracasei* 19, L. *acido* La5, *Bifido lactis* Bb12; **6 strains**, “LacClean®” L. *acidophilus* 11906BP, L. *rhamnosus* 12202BP, *Bifido. bifidum* 12199BP, *Bifido. lactis* 11,904 BP, *Bifido. longum* 12,200 BP, *Strept. thermophilus* 11870BP; **7 strains**, “DuoLac®*: Bifido. brevis* 11858BP, *Bifido. lactis* 11903BP, *Bifido. longum* 11860BP*,* L. *acidophilus* 11906BP, L. *rhamnosus* 11868BP, L. plantarum 11867BP, *Strept. thermophilus* 11870BP; **8 strains**, *Bifido. breve* DSM24732, *Bifido. longum* DSM24736, *Bifido. infantis* DSM24737, L. *acidophilus* DSM24735, L. *plantarum* DSM24730, L. *paracasei* DSM24733, L. *delbruckii* subsp. *bulgaricus* DSM24734, *Strept. thermophiles* DSM24731, originally named VSL#3®, now either Visbiome™ or Vivomixx™ using the De Simone formulation.

#### 3.6.3 Change in abdominal pain scores

Of the 14 different probiotic types, 12 had at least two RCTs/type for this outcome. This was the most commonly reported IBS outcome in all the 40 RCTs. Four probiotics showed a significant reduction in abdominal pain scores (Fig. 3): *Bac. coagulans* MTCC5260 (SMD= −1^.^7, 95% C.I. −2^.^3, −1^.^1, *P* < 0.001), *L. rhamnosus* GG (SMD= −3^.^3, 95% C.I. −5^.^65, −1^.^1, *P* = 0.003), a 7-strain mixture (SMD= −5^.^4, 95% C.I. −8^.^9, −1^.^8, *P* = 0.003), and an 8-strain mixture (SMD= −1^.^7, 95% C.I. −3^.^3, −0^.^01, *P* = 0.049), while a non-statistically significant trend was seen for *S. boulardii* CNCM I-745 (*P* = 0.09), a 6-strain mixture (*P* = 0.07) and *Bac. coagulans* MTCC5856 (*P* = 0.07). No significant reduction in pain scores were seen for the other five probiotics. Overall, the heterogeneity in the 29 RCTs with this outcome was high (I^2^= 95.9%), but by stratifying by probiotic type, low heterogeneity (from I^2^= 0–39%) was achieved for 2/12 (17%) of the probiotic sub-groups.

#### 3.6.4 Frequency of abdominal pain relief

**Fig. 4:**
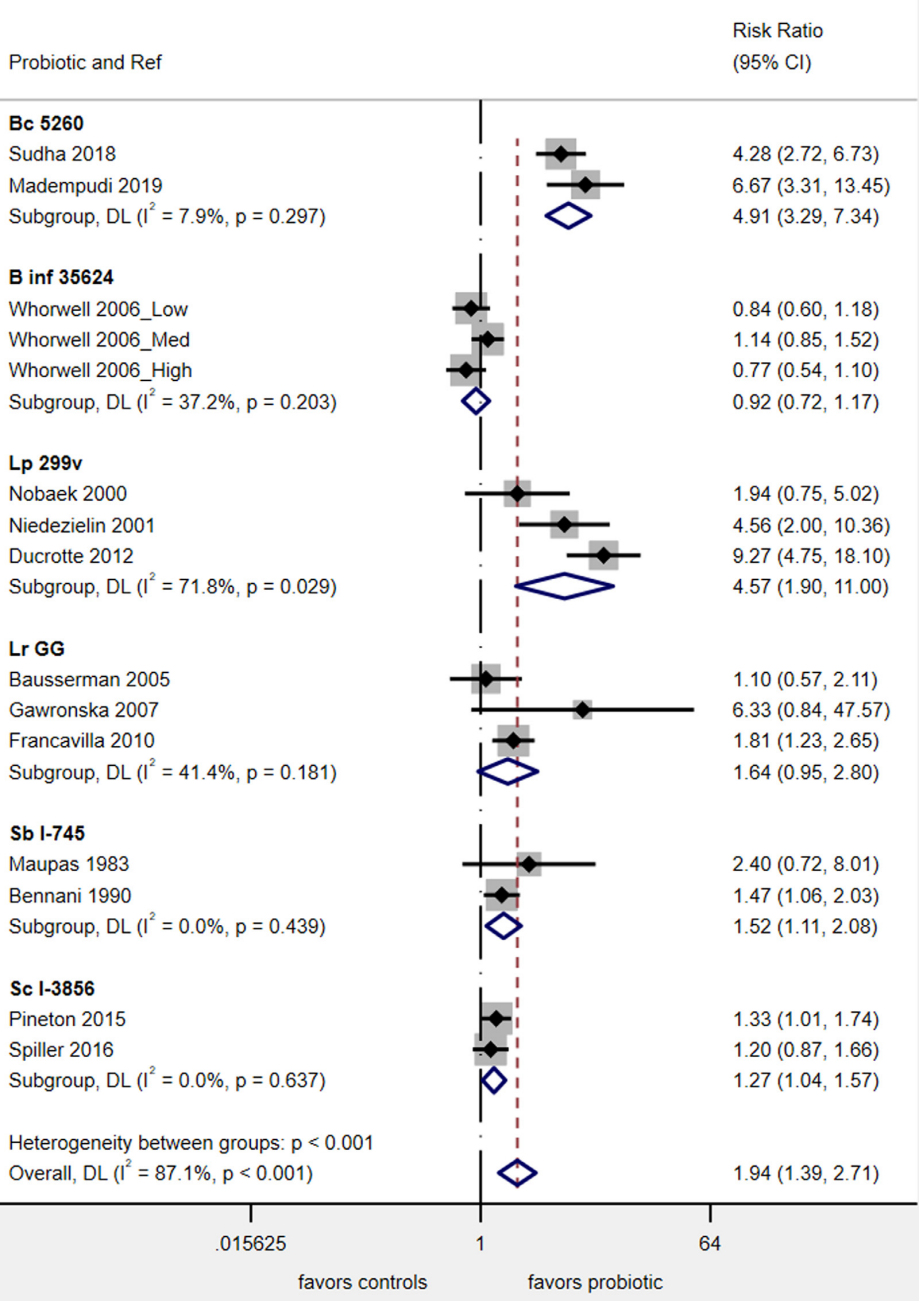
Forest plot of probiotics for frequency reporting less abdominal pain by study end. Boxes indicate each study's relative risk and horizontal lines indicate each study's 95% confidence intervals, Diamond indicates subgroup's pooled RR and 95% C.I. **Abbreviations**: **Bc**, *Bacillus coagulans*; **B inf,***Bifidobacterium infantis*; L., *Lactobacillus*; Lp, L. *plantarum*; **Lr***,* L. *rhamnosus*; **Sb**, *Saccharomyces boulardii*; ***Sc***, *Saccharomyces cerevisiae.*

Six of the 14 probiotic types had at least two RCTs for this outcome (Fig. 4). Four probiotics significantly improved the report of abdominal pain by the end of the study: *Bac. coagulans* MTCC5260 (RR= 4^.^9, 95% C.I. 3^.^3, 7^.^3, *P* < 0.001), *L. plantarum* 299v (RR= 4^.^6, 95% C.I. 1^.^9, 11^.^0, *P* = 0.001), *S. boulardii* CNCM I-745 (RR= 1^.^5, 95% C.I. 1^.^1, 2^.^1, *P* = 0.009) and *S. cerevisiae* CNCM I-3856 (RR= 1^.^3, 95% C.I. 1^.^04, 1^.^6, *P* = 0.02). *L. rhamnosus* GG had a non-statistically significant trend (*P* = 0.07) but *Bif. infantis* 35624 had no significant impact on abdominal pain relief. Overall, the heterogeneity in the 13 RCTs with this outcome was high (I^2^=87%), but by stratifying by probiotic type, low heterogeneity (from I^2^= 0–37%) was achieved for 5/6 (83%) of the probiotic sub-groups.

#### 3.6.5 Change in bloating severity scores

Only nine RCTs (10 treatment arms) measured the change in bloating severity scores. Two single strain probiotics and two multi-strain mixtures were eligible for this analysis, but none demonstrated significant efficacy for this outcome (Supplementary appendix Fig. 4). Overall, the heterogeneity in the 9 RCTs with this outcome was high (I^2^= 87%), but by stratifying by probiotic type, low heterogeneity (from I^2^= 0–6%) was achieved for all 4 (100%) of the probiotic sub-groups.

### 3.7 Sub-group analyses

*IBS sub-types*. Most RCTs enrolled a mixed population of different of IBS subtypes, but failed to analyze outcomes by specific IBS subtypes. Of the 42 RCTs, only 14 (33%) reported outcome data separately by IBS subtypes, either as sub-groups in mixed types of enrolled IBS patients [24,37,45,46,50,53] or only enrolled patients with IBS-D [22,42,43,[54], [55], [56]] or only IBS-C [25,26]. Only 8 RCTs with common outcomes and at least two RCTs/type could be analyzed. Of the 4 RCTs IBS-D patients [42,43,54,55] two probiotics were analyzed and only the 7-strain mixture significantly reduced abdominal pain scores (SMD= −5^.^8, 95% C.I. −9^.^7, −1^.^9, *P* = 0.004), as shown in Supplementary appendix Fig. 5. Stratifying by probiotic strain types reduced overall heterogeneity for the four IBS-D trials from I^2^= 96.7% to 0%, but only for *S. boulardii* I-745. Of the 4 RCTs in IBS-C patients,[25,26,45,46] neither *S. cerevisiae* CNCM I-3856 nor *Bif. animalis* DN173010 had a significant reduction in abdominal pain scores, as shown in Supplementary appendix Fig. 6. Stratifying by probiotic strain types reduced overall heterogeneity for the four IBS-C trials from I^2^=48.5% to 0%, but only for *Bif. animalis* DN173010.

*Daily dose of probiotic*. Overall, in the 45 treatment study arms, 16 (36%) used the probiotic at a dose of 10^6^–10^9^ cfu/day, 23 (51%) used a higher dose (10^10^/day), five (11%) used the highest dose at 10^11^/day, while one study did not report the daily dose used [28]. Once we stratified on the type of probiotic and a common outcome, our analysis was limited due to either low numbers of trials or similar daily doses used within the same type of probiotic. Only two types of probiotics were tested with different daily doses*: Bif. infantis* 35624 (10^6^, 10^8^ and 10^10^) [24] and the 4-strain mixture (10^9^) [50] and (10^10^) [51]. A dose-response was only seen for the 10^8^ dose of *Bif. infantis* 35624 and the 10^10^ dose of the 4-strain mixture (Table 1).

*Adult* versus *pediatric patients* The majority of the study participants were adults (86% of the trials) and only six RCTs were done in children assessing either *Bac. coagulans* 5260 [18] or *L. rhamnosus* GG [[34], [35], [36],^38^] or the 8-strain mixture [58]. Using the most common IBS outcome (change in abdominal pain scores), the reduction in pain scores appeared to be similar in adult and pediatric trials (data not shown), but we were unable to conduct a strain-specific meta-analysis by type as there was only one trial in adults and one in children for each of the two the probiotic types (*B. coagulans* 5260 and the 8-strain mixture) and there were no trials done in adults with a common IBS outcome to compare to the four pediatric trials using *L. rhamnosus* GG.

## 4 Discussion

To the best of our knowledge, this is the first meta-analysis that accounted for probiotic strain-specificity when assessing efficacy for the treatment of IBS. Overall, this study showed six single-strain probiotics (*Bac. coagulans* MTCC5260, *Bif. infantis* 35624, *L. plantarum* 299v, *L. rhamnosus* GG, *S. boulardii* CNCM I-745 and *S. cerevisiae* CNCM I-3856) and three mixtures (a 4-strain mixture, a 7-strain mixture and an 8-strain mixture) showed significant improvement in at least one IBS outcome measure. As the choice of an appropriate type of probiotic can be challenging for clinicians and policy-makers [5], this analysis offers nine probiotics supported by rigorous analyses following recommended guidelines for strain-specificity and having at least two RCTs per probiotic type [7,8].

This review has several strengths, in that it was an extensive and broad-based literature review. The trial selection, data extraction and quality scoring was done independently by multiple researchers to minimize bias and transcription errors. The meta-analysis accounted for both strain-specific assessment of probiotics and type of outcome measures. Another strength is that most included trials were of moderate-high quality that had at least one confirmatory trial using the identical probiotic strain or strains.

The review does have several limitations. Of 64 different types of probiotics screened, 50 had insufficient numbers of randomized trials (*n* < 2) to be included in this analysis. We cannot conclude these 50 probiotic types may not be effective for IBS patients, rather we just lacked confirmatory trials to form a robust conclusion of efficacy. These 50 types of probiotics may be potential candidates for IBS, but more RCTs are required for these strains. IBS trials also had diverse outcomes used to measure efficacy: responders (usually improved symptoms), changes in symptom severity scores, quality of life, stool consistency, etc. and different scales and score ranges used by different studies hampered the comparisons of a common outcome measure. Studies often had a heterogeneity of IBS sub-groups included or failed to report outcome efficacy by IBS sub-types, thus limiting analysis by IBS sub-types. Variable study designs (different doses, varying duration of treatments, lack of adequate follow-up to determine if the effect was sustainable, lack of adverse event data) made it difficult to determine the role of daily probiotic dose and duration for the various probiotics. The formulation of the probiotic was also diverse and fermented milk products may have prebiotic effects, which were not addressed in the trials. The generalizability of these results is also limited, as most of the trials were done with adults and there was no documentation of other risk factors (such as diet), which may impact IBS symptoms.

Probiotics have been investigated as IBS treatments but as the efficacy of probiotics has been determined to be strain-specific [6], previous meta-analyses of IBS may have reached erroneous conclusions if the strain-specificity was not accounted for. An extensive systematic review of 66 RCTs of probiotics, prebiotics, synbiotics and antibiotics for the treatment of IBS could not conclude which treatments might be more effective due to significant heterogeneity between studies and different types of treatments [9]. Even recent meta-analyses have inappropriately pooled different types of probiotic strains at a genus level (that is, pooling all trials of any type of *Lactobacilli* or *Bifidobacterium* strains into genus-level groups) and then concluding all *Lactobacilli* or all *Bifidobacteria* may be effective or not [3,9,60,61]. One meta-analysis included both trials of IBS patients and non-IBS patients with chronic constipation (but no other Rome criteria) and based their conclusions on this mixed group of diseases and also incorrectly pooled different types of probiotics together [10]. More recent meta-analyses focused on including a single type of probiotic in an effort to account for strain-specificity. Yuan et al. pooled six RCTs that used *Bifidobacterium infantis* 35624, but they erroneously included two trials of different probiotic mixtures that used a different strain of *Bifidobacteria* [62,63]. Another meta-analysis limited included trials to those testing *S. cerevisiae* CNCM I-3856, but found only two RCTs with inconsistent results [64]. Other meta-analyses of probiotics for IBS have also included probiotics with a single trial, pooling this data into a larger dataset of all probiotic strains. The British Society of Gastroenterology published their 2021 guidelines for the treatment of IBS and recommended probiotics, in general, as a first line treatment, but as their analysis pooled dissimilar strains of probiotics, they could not identify which specific probiotics might be useful [3]. This meta-analysis might help to overcome this limitation.

Controversies exist for the use of probiotics, mainly including which probiotics have proven efficacies. This has been addressed by limiting our analysis to probiotic types with sufficient number of RCTs and common IBS outcomes. Future trials are needed for other probiotic candidates currently supported by only a single trial. Another issue is what role dead or heat-modified bacterial or fungal microbes or prebiotics may have for the efficacy of disease. As the efficacy of modified microbes is debated, [65], [66], [67] we limited our analysis to living probiotics, as this follows the international consensus that probiotics must be alive [13].

For future studies, there is a need to have standardized IBS outcomes, using at least one global improvement measure, so that different trial results may be compared. In addition, many studies used different scales with varied ranges of values, making comparisons between studies difficult. Future reviews and meta-analysis need to analyze probiotics separately by the type of probiotic and have at least one confirmatory trial for each outcome. As IBS criteria for diagnosis were revised in 2016 as Rome IV and future studies should use this basis for IBS diagnosis and to report efficacy data by the IBS subtypes. Acidified or fermented milk usage as probiotic form causes prebiotic effect, so capsule or granule forms should be advocated. Exclusion criteria should be expanded because of comorbidities may affect the study, such as small intestinal bacterial overgrowth. Synchronous prebiotic or over the counter usage should be restricted to demonstrate actual probiotic effect. As safety of investigational treatments is an important consideration, future studies need to report adverse event data in more detail. As we found 17% of the trials in our review did not report any safety data, it is important to fully document safety data. The paucity of adverse event data published in probiotic trials should be rectified in future studies, as this is an important clinical consideration. As 50 types of probiotics did not have a confirmatory trial, this opens an opportunity for future research to identify more potential probiotics for the treatment of IBS. As research defining microbiome profiles continues, it may be possible to determine if different microbiome profiles respond better to specific probiotic types or if individual microbiome profiles may be helpful in determining treatment responses for IBS patients.

Although the analysis of probiotics was limited by the diversity of IBS outcomes used in trials, six single-strain probiotics and three different types of probiotic mixtures showed significant efficacy for at least one IBS outcome measure.

## Funding

This study was unfunded.

## Contributors

LVM designed the study; LVM oversaw the conduct of the study; LVM designed and conducted the analysis; LVM designed and conducted the statistical analysis; LVM, TK and AK independently reviewed and scored all reviewed papers and all had access to all data; LVM, TK and AK wrote the manuscript and all authors reviewed and contributed to the final manuscript. LVM, TK and AK all verified the underlying data.

## Data sharing

Data collected for this meta-analysis have already been published in other studies. Data extracted from these published articles are available in the supplementary materials. Protocol and statistical analysis available at: https://www.crd.york.ac.uk/PROSPERO (CRD42018109169). There are no individual participant data due to the nature of this meta-analysis. The data will be made available upon publication and ending 5 years following article publication.

## Declaration of Competing Interest

LVM is on the Scientific Advisory Board of Bio-*K*+ (Bio-K+, a Kerry company) and on the Biocodex Microbiome Board (Biocodex, France) and has received honoraria from Bio-K+ and Biocodex. TK and AK declared no conflicts of interest.

## Appendix Supplementary materials

Image, application 1

Image, application 2
