# Supplementary material for: Strain-specific and outcome-specific efficacy of probiotics for the treatment of irritable bowel syndrome: A systematic review and meta-analysis
Source: eClinicalMedicine. 2021 Oct 18;41:101154. doi: 10.1016/j.eclinm.2021.101154 (PMC8529205; doi:10.1016/j.eclinm.2021.101154)
Supplement: Supplementary file 1 [file mmc1.docx]

**Captions for Supplementary Material**

**Text 1. Study Protocol**

**Table 1.** **Excluded randomized controlled trials (n=45) in IBS patients treated with probiotics.**

**Table 2. Study population and intervention characteristics in 42 randomized controlled trials in IBS patients treated with either probiotics or controls.**

**Table 3.** **Adverse reactions and safety in 42 randomized controlled trials in IBS patients treated with either probiotics or controls.**

**Figure 1. Risk of bias in individual included trials of IBS and probiotics.**

**Figure 2. Funnel plot of publication bias.**

**Figure 3. Forest plot of frequency of “Responders” by probiotic type.**

**Figure 4. Forest plot of change in bloating scores in IBS patients treated with probiotics or controls.**

**Figure 5. Forest plot of change in abdominal pain scores in patients with IBS-D only treated with probiotics or controls.**

**Figure 6. Forest plot of change in abdominal pain scores in patient with IBS-C only treated with probiotics or controls.**
