# Supplementary material for: Strain-specific and outcome-specific efficacy of probiotics for the treatment of irritable bowel syndrome: A systematic review and meta-analysis
Source: eClinicalMedicine. 2021 Oct 18;41:101154. doi: 10.1016/j.eclinm.2021.101154 (PMC8529205; doi:10.1016/j.eclinm.2021.101154)
Supplement: Supplementary file 2 [file mmc2.docx]

**Supplementary Material: Text 1. Study Protocol**

Strain-specific and outcome-specific efficacy of probiotics for the treatment of Irritable Bowel Syndrome: A systematic review and meta-analysis

**Lynne V. McFarland**, PhD, M.S., Affiliate Associate Professor, Dept. Medicinal Chemistry, School of Pharmacy, University of Washington, Seattle, Washington 98195 USA, Email: [mcfarland.lynne.v@gmail.com](mailto:mcfarland.lynne.v@gmail.com) phone: 1-206-526-5729.

**Tarkan Karakan,** M.D. Proffesor, Deparment of Gastroenterology, Gazi University Faculty of Medicine, 06500, Beşevler, Ankara, Turkey

**Ali Karatas**, M.D Deparment of Gastroenterology, Gazi University Faculty of Medicine, 06500, Beşevler, Ankara Turkey

**INTRODUCTION**

Irritable bowel syndrome (IBS) is a chronic functional gastrointestinaI disorder characterized by recurrent abdominal pain, bloating and changes in bowel habits. IBS affects 15-20% of adults in developed countries with higher prevalence in developing countries. Symptoms are using to classify sub-types of IBS: diarrhea-predominant IBS (IBS-D), constipation-predominant IBS (IBS-C) or mixed/alternating symptoms (IBS-M). Major risk factors for IBS include female gender, family history of IBS, environmental triggers such as changes in diet or lifestyle or stress.^1,2^ The pathophysiology of IBS involves chronic mucosal inflammation, alterations in intestinal epithelial and immune functions, visceral hypersensitivity, increased intestinal permeability and dysbiosis of the intestinal microflora. Current guidelines recommend medications focused on IBS symptoms, diets low in fermentable types of oligosaccharides, rifaximin and the use of probiotics.^3,4^ Recent meta-analyses have failed to determine which type of probiotics may be more effective due to inappropriate pooling of dissimilar types of probiotics^5-7^ or the inclusion of non-IBS functional constipation studies.^8^ As the efficacy for probiotics is both disease-specific and strain-specific,^10^ there is a need to account for both of these factors when providing guidance for IBS patients. The aim of this study well be to determine which probiotic strains are safe and effective for the treatment of IBS.

***MATERIALS AND METHODS***

***Search strategy and study selection***

This review is an update from a prior meta-analysis on IBS, but will include trials and recommendations published in the subsequent 13 years.^1^ PubMed, Google Scholar and NIH registry of clinical trials will be searched from database inception to June 2021, unrestricted by language or year of publication. EMBASEW and SCOPUS will also be reviewed if necessary. Non-English papers will be translated. Proposed search strategy was as follows: (“probiotics” [MeSH Terms] OR “probiotics” [All Fields]) AND “irritable bowel syndrome” [MeSH Terms] OR “irritable bowel syndrome” [All Fields] AND “controlled trials”). We will also search for IBS subtypes. Additional searches will be done using known probiotic types. Secondary searches of grey literature will include reference lists, authors, reviews, meeting abstracts websites and clinicaltrials.gov for unpublished trials. A recursive search will be performed, using the bibliographies of all obtained articles.

Inclusion criteria include: randomized, controlled clinical trials (RCTs), blinded or open interventions, adult or pediatric patients and published in peer-reviewed journals. We will include only probiotics fulfilling the standard definition (must be living microbe, of adequate dose and having efficacy for a health effect.^10^ This definition excludes dead or heat-killed microbes and prebiotics. Each probiotic type (single strain or multi-species mixture) will be required to have at least two RCTs per type sharing at least one common IBS outcome. As bacterial and fungal taxonomies shift over time, the most current strain designations will be presented in this review and strain identification will be confirmed with the original authors or the manufacturer whenever possible. Exclusion criteria include: non-human studies, early phase 1 or 2 safety or mechanism of action studies, no control group, probiotic not well described, reviews and duplicate reports. Current recommendations require that each type of probiotic be analyzed as a separate sub-group and not to pool dissimilar types of probiotics.^9,11^ We will follow this recommendation and only pooled identical types of probiotic strains or mixtures of the same strains when analyzing efficacy for IBS. The protocol was registered with Prospero (Prospero #: CRD42018109169).

***Data extraction***

Initial screening of studies and data extraction will be done independently by one author (LM), then independently reviewed by one of the other two co-authors (TK or AK) following the standard methods for systematic reviews and meta-analysis.^12^ Any disagreements will be discussed until consensus is reached. The data extracted from each study will use a standard data extraction form for PICOS data: (1) patient population (adult/pediatric, age, country of travel origin, type of IBS), (2) intervention (type of probiotic or controls used, daily doses, formulation, duration and follow-up times), (3) comparisons (type of control group either placebo or open, unblinded), (4) IBS outcomes reported and (5) study design (randomized, controlled trials, either double blinded or open). For data that are required for these analyses but not reported in the published article, we will attempt to contact the author or co-authors to obtain the missing data.

***Outcomes assessment***

As no consensus has been reached for a standardized outcome to evaluate IBS improvements, we will include the most common outcomes found in the included trials. The primary outcomes that were screened included: (1) “Change in global IBS-SSS scores”, a continuous outcome comparing the change of overall symptom scores from baseline to end of study; (2) “Frequency of Responders”, a dichotomous outcome defined as improvement of global IBS symptoms reported by the end of the study (either by physician assessment or by subject interview or diaries); (3) “Change in abdominal pain scores”, a continuous outcome comparing the change of scores for abdominal pain from baseline to end of study; (4) “Frequency reporting abdominal pain relief”, a dichotomous outcome (either by physician assessment or by subject interview or diaries) by the end of the study; (5) “Change in bloating scores”, a continuous outcome comparing the change in scores for bloating from baseline to end of study and (6) “Change in QoL (quality of life) scores”, a continuous outcome comparing the change of scores for quality of life from baseline to end of study. The secondary outcome will be the number of adverse reactions by group.

***Assessment of study quality and risk of bias***

Each included RCT will be reviewed and scored independently by at least two of the three co-authors using standard methods.^12^ The risk of bias will be graded (high, low or not reported) for each of six types of bias [selection bias (method of randomization and blinded allocation), performance bias (degree of blinding of study personnel and study subjects), detection bias (outcome assessor blinded), attrition bias (attrition different by group), reporting bias (*a priori* outcomes reported) and other issues (fraud or miscellaneous)].^13^

***Data synthesis and statistical methods***

We will use the standard PRISMA (Preferred Reporting Items for Systematic reviews and Meta-analysis) guidelines for this review,^12^ but will follow more recent recommendations to account for probiotic strain specificity.^9,11^ Statistical analysis and generation of forest plots of pooled summary estimates was performed using Stata software version 16 (Stata Corporation, College Station, Texas) with meta-analysis modules.^14^ Summary estimates will be based on the pooled data from RCTs using the same type (species) of probiotic and sharing a common IBS outcome measure. Dichotomous outcomes will be assessed using relative risks (RR) and 95% confidence intervals (C.I.) and continuous outcomes will be assessed using standard mean difference (SMD) and 95% C.I. using standard methods.^15^ Heterogeneity across trials will be evaluated using the I^2^ statistic.^16^ Random effects models will be used for the meta-analysis if heterogeneity was found (I^2^>50% for overall effect) or to allow for differences across studies. Publication bias will be assessed using the Egger test.^17^ *A priori* sub-groups will include: sub-type of IBS (IBS-C, IBS-D or IBS-M) and daily doses of probiotic used (<10^9^/day or >10^9^/day), type of study population (adult/pediatric/mixed) and degree of study bias.

**References**

1. McFarland LV, Dublin S. Meta-analysis of probiotics for the treatment of irritable bowel disease. *World J Gastroenterol.* 2008; 14(17):2650-61.

2. Van den Houte K, Carbone F, Pannemans J, et al. Prevalence and impact of self-reported irritable bowel symptoms in the general population. *United Euro Gastroenterol J.* 2019;7(2):307-315. [doi: 10.1177/2050640618821804]

3. Vasant DH, Paine PA, Black CJ, et al. British Society of Gastroenterology guidelines on the management of irritable bowel syndrome. *Gut* 2021; 70(7):1214-124-. [doi: 10.1136/gutjnl-2021-324598].

4. Altobelli E, Del Negro V, Angeletti PM, Latella G. Low-FODMAP Diet Improves Irritable Bowel Syndrome Symptoms: A Meta-Analysis. *Nutrients*. 2017; 9(940):1-19. [doi: 10.3390/nu9090940]

5. Ford AC, Harris LA, Lacy BE, Quigley EMM, Moayyedi P. Systematic review with meta-analysis: the efficacy of prebiotics, probiotics, synbiotics and antibiotics in irritable bowel syndrome. *Aliment Pharmacol Ther*. 2018;48(10):1044-1060. [doi: 10.1111/apt.15001]

6. Liang D, Longgui N, Guoqiang X. Efficacy of different probiotic protocols in irritable bowel syndrome: A network meta-analysis. *Medicine* 2019;98:27:e16068.

7. Niu H, Xiao J. The efficacy and safety of probiotics in patients with irritable bowel syndrome: Evidence based on 35 randomized controlled trials. *Intl J Surg* 2020;75:116-127.

8. Wen Y, Li J, Long Q, Yue C, He B, Tang X. The efficacy and safety of probiotics for patients with constipation-predominant irritable bowel syndrome: A systematic review and meta-analysis based on seventeen randomized controlled trials. *Intl J Surg* 2020; 79:111-119.

9. McFarland LV, Evans CT, Goldstein EJC. Strain-specificity and disease-specificity of probiotic efficacy: a systematic review and meta-analysis. *Frontiers in Medicine* 2018: 5 (124):1-14. [doi: 10.3389/fmed.2018.00124]

10. Hill C, Guarner F, Reid G, et al. Expert consensus document: The International Scientific Association for Probiotics and Prebiotics consensus statement on the scope and appropriate use of the term probiotic. *Nat Rev Gastroenterol Hepatol*. 2014; 11(8):506-14. [doi: 10.1038/nrgastro.2014.66]

11. Szajewska H. Pooling data on different probiotics is not appropriate to assess the efficacy of probiotics. *Eur J Pediatr.* 2014;173(7):975. [doi: 10.1007/s00431-014-2340-4]

12. Moher D, Shamseer L, Clarke M, et al. Preferred reporting items for systematic review and meta-analysis protocols (PRISMA-P) 2015 statement. *Syst Rev.* 2015;4:1. [doi 10.1186/2046-4053-4-1]

13. McGuinness LA. Robvis: An R package and web application for visualising risk-of-bias assessments, 2019. Available at: https://github.com/mcguinlu/robvis. Accessed June 3, 2019.

14. Palmer TM and Sterne JAC (ed.) Meta-analysis in Stata: an updated collection from the Stata Journal. Second Edition. 2016. Published by Stata Press, College Station, Texas.

15. Der Simonian R, Laird N. Meta-analysis in clinical trials. *Control Clin Trials.* 1986;7(3):177-88.

16. Ioannidis JP. Interpretation of tests of heterogeneity and bias in meta-analysis. *J Evaluation Clin Prac*. 2008;14(5):951-7.

17. Egger M, Davey-Smith G, Schneider M, Minder C. Bias in meta-analysis detected by a simple, graphical test. *BMJ.* 1997; 315:629-634.

**Supplementary Table 1. Excluded randomized controlled trials (n=45, 50 treatment arms) in IBS patients treated with probiotics**

| **Reference** | **Probiotics with only one trial/type** |
| --- | --- |
| Amirimani B 2013^1^ | “Biogaia*” L. reuteri* DSM17938 |
| Andriulli A 2008^2^ | *L. paracasei* B21060 |
| Basturk A 2016^3^ | *Bifido. lactis* B94 |
| Cappello C 2013^4^ | 9 strains “Probinl” synbiotic: *L. acido, L. plantarum, L. rhamnosus, L. gasseri, L. salivarus, L. sporogenes, Bifido. infantis* B102, *Bifido. longum, Strept thermophilus+* FOS |
| Choi SC 2011^5^ | 3 strains*: L. acido, L. infantis, Strept. thermophilus* |
| Cui S 2012^6^ | 3 strains “Bifido Triple Viable”: *L. acidophilus* nr, *Bifido. longum* nr, *Enterococcus faecalis* nr |
| Dapoigny M 2012^7^ | *L. casei rhamnosus* LCR35 |
| Drouault-Holowacz S 2008^8^ | 4 strains “Lactibiane”: *Bifido .longum* LA101, *L. acidophilus* LA102, *L. lactis* LA103, *Strept. thermophilus* LA104 |
| Enck P 2008^9^ | *E. coli* DSM17252, *Entero. faecalis* DSM 16440 |
| Enck P 2009^10^ | *E. coli* 17252 |
| Fanigliulo L 2006^11^ | *Bifido. longum* W11 |
| Francavilla R 2018^12^ | 5 strains: *L. casei* LMG 101/37 P-17504, *L. plantarum* CECT 4528, *Bifido. animalis lactis* Bi1 LMG P-17502, *Bifido. breve* Bbr8 LMG P-17501, *Bifido. breve* B110 LMG P-17500 |
| Gade J 1989^13^ | *Entero. faecalis* 40371 |
| Giannetti E 2017^14^ | *Bifido. infantis* M63, *Bifido. breve* M16V*, Bifido. longum* BB536 |
| Guglielmetti S 2011^15^ | *Bifido. bifidum* MIMBb75 |
| Gupta AK 2021^16^ | *Bacillus coagulans* LBSC |
| Hod K 2017^17^ | 11 strains “Bio25”: *L. acidophilus* LA1, *L. casei* LC5, *L. rhmanosus* LR5*, L. paracasei* LPC5, *L. plantarum* LP3, *Bifido. bifidum* BF3, *Bifido. longum* BG7, *Bifido. breve* BR3, *Bifido. infantis* BT1, *Lactococcus lactis* SL6, *Strept thermo* ST3 |
| Hong KS 2009^18^ | 4 strains: *Bifido. bifidum* BGN4, *Bifido. lactis* AD011, *L*. *acidophilus* ADO31, *L. casei* IBS041 |
| Ishaque SM 2018^19^ | 14 strain mixture “Bio-Kult”: *Bac. subtilis* PXN21, *Bifido. bifidum* PXN23, *Bifido. breve* PXN25, *Bifido. infantis* PXN27, *Bifido. longum* PXN30, *L. acidophilus* PXN35, *L. bulgaricus* PXN39, *L. casei* PXN37, *L. plantarum* PXN47, *L. rhamnosus* PXN54, *L. helveticus* PXN45, *L*. *salivarius* PXN57, *Lactococcus lactis* PSN63, *Strept. thermophilus* PPXN66. |
| Jafari E 2014^20^ | “4 strains Probio-Tec” : *Bifido. animalis* Bb12, *L. acidophilus* La5,  *L. delbruckii bulgaricus* 27, *Strept. thermophilus* STY31 |
| Khodadoostan M 2018^21^ | 7 strains “Familact” synbiotic: *L. casei, L. acidophilus, L. rhamnosus, L. bulgaricus, Bifido. breve, Bifido. longum, Strept. thermophilus* + FOS |
| Kim YG 2006^22^ | 2 strains “Medilac DS”: *Bac. subtilis* RO179, *Strept. faecium* |
| Lewis ED 2020^23^ | *Lactobacillus paracasei* HA-196 arm *Bifidobacterium longum* R0165 arm |
| Lorenzo V 2014^24^ | 3 strains: *L. plantarum* CECT7484, *L. plantarum* CECT7485, *Pediococcus acidilactic* CECT7483 |
| Ludidi 2014^25^ | 6 strains “Winclove 801”: *Bifido. lactis* W52, *L. casei* W56, *L. salivarius* W57, *L. acidophilus* NCFT, *L. rhamnosus* W71, *L. lactis* W58 |
| Lyra A 2016^26^ | *L. acidophius* NCFM |
| Martoni CJ 2020^27^ | *Bifidobacterium longum* R0175 arm *L. paracasei* HA196 arm |
| Mezzasalma V 2016^28^ | *L acidophilus* DSM 24936 arm *L. reuteri* 25175 arm |
| Murakami K 2012^29^ | *L. brevis* KB290 |
| Niv E 2005^30^ | *L. reuteri* 55730 |
| Oh JH 2019^31^ | 3 strains: *L. paracasei, L. salivaricus, L. plantarum* |
| O’Mahony L 2005^32^ | *L. salivaris* UCC 4331 arm |
| Preston K 2018^33^ | 3 strains “BioK+”: *L. acidophilus* CL1285, *L casei* LBC80R, *L*. *rhamnosus* CLR2 |
| Ringel Y 2011^34^ | *L. acidophilus* NCFM, *Bifido. lactis* Bi07 |
| Roberts LM 2013^35^ | *Bifido. lactis* DN173010 (CNCM I-2494) |
| Sadrin S 2020^36^ | 2 strains: *L. acidophilus* ATCC SD5221*, L. acidophilus helveticus* LAFT1 |
| Saggioro A 2004^37^ | *L. acidophilus* LAD*, L. plantarum* LP01 |
| Shavakhi A 2013^38^ | 7 strains: “Protexin” “Balance” *L. acidophilus, L. casei, L. rhamnosus, L. bulgaricus, Bifido. breve, Bifido. longum, Strept thermophilus*, FOS |
| Shin SP 2018^39^ | *L. gasseri* BNR17 |
| Sinn DH 2008^40^ | *L. acidophilus* SDC 2012, 2013 |
| Sisson G 2014^41^ | 4 strains “Symprove”: *L. rhamnosus* 30174, *L. plantarum* 30173, *L*. *acidophilus* 30175, *Entero. faecalis* 30176 |
| Skrzydio-Radmanska B 2020^42^ | 5 strain synbiotic: *L. rhamnosus* 19070-2, *L. acidophilus* DSMZ32418, *Bifido. lactis* DSMZ32269, *Bifido. longum* DSMZ32946, *Bifido. bifidum* DSMZ32403 + FOS |
| Sun YY 2018^43^ | *Clost. butyricum* |
| Thijssen AY 2016^44^ | *L. casei* Shirota |
| Williams EA 2009^45^ | 4 strains “LAB4”: *L. acidophilus* CUL60, *L. acidophilus* CUL21, *Bifido. lactis* CUL34, *Bifido. bifidum* CUL20 |
| Zeng J 2008^46^ | 4 strains “AB100”: *L. acidophilus* nr, *L. bulgaricus* nr, *Bifido. longum* nr, *Strept thermophilus* nr |

**Abbreviations**: Bac., Bacillus; Bifido., Bifidobacterium; Clost., Clostridium; E., Escherichia; Entero., Enterococcus; L., Lactobacillus, nr, strain not reported in paper; RCT, randomized controlled trial; Strept, Streptococcus

**Excluded IBS references**

1. Amirimani B, Nikfam S, Albaji M, et al. Probiotic vs. placebo in irritable bowel syndrome: a randomized controlled trial. *Middle East J Dig Dis* 2013;5(2): 98-102.

2. [Andriulli A](http://www.ncbi.nlm.nih.gov/pubmed/?term=Andriulli%20A%5BAuthor%5D&cauthor=true&cauthor_uid=18685503), [Neri M](http://www.ncbi.nlm.nih.gov/pubmed/?term=Neri%20M%5BAuthor%5D&cauthor=true&cauthor_uid=18685503), [Loguercio C](http://www.ncbi.nlm.nih.gov/pubmed/?term=Loguercio%20C%5BAuthor%5D&cauthor=true&cauthor_uid=18685503), et al. Clinical trial on the efficacy of a new symbiotic formulation, Flortec, in patients with irritable bowel syndrome: a multicenter, randomized study. [*J Clin Gastroenterol*](http://www.ncbi.nlm.nih.gov/pubmed/?term=andriulli+A+and+IBS) 2008;42 Suppl 3 Pt 2:S218-23. [doi: 10.1097/ MCG.0b013e31817fadd6]

3. [Baştürk A](https://www.ncbi.nlm.nih.gov/pubmed/?term=Ba%C5%9Ft%C3%BCrk%20A%5BAuthor%5D&cauthor=true&cauthor_uid=27782892), [Artan R](https://www.ncbi.nlm.nih.gov/pubmed/?term=Artan%20R%5BAuthor%5D&cauthor=true&cauthor_uid=27782892), [Yılmaz A](https://www.ncbi.nlm.nih.gov/pubmed/?term=Y%C4%B1lmaz%20A%5BAuthor%5D&cauthor=true&cauthor_uid=27782892). Efficacy of synbiotic, probiotic, and prebiotic treatments for irritable bowel syndrome in children: A randomized controlled trial. [T*urk J Gastroenterol*](https://www.ncbi.nlm.nih.gov/pubmed/?term=IBS+and+Basturk) 2016 Sep;27(5):439-443. [doi: 10.5152/tjg.2016.163017 strains “Familact” synbiotic: *L. casei, L. acidophilus, L. rhamnosus, L. bulgaricus, Bifido. breve, Bifido. longum, Strept. thermophilus* + FOS

4. Cappello C, Tremolaterra F, Pascariello A, Ciacci C, Iovino P. [A randomised clinical trial (RCT) of a symbiotic mixture in patients with irritable bowel syndrome (IBS): effects on symptoms, colonic transit and quality of life.](https://www.ncbi.nlm.nih.gov/pubmed/22885882) *Int J Colorectal Dis* 2013 Mar;28(3):349-58. [doi: 10.1007/s00384-012-1552-1, PMID: 22885882]

5. Choi SC, Kim BJ, Rhee PL, et al. [Probiotic Fermented Milk Containing Dietary Fiber Has Additive Effects in IBS with Constipation Compared to Plain Probiotic Fermented Milk.](https://www.ncbi.nlm.nih.gov/pubmed/21461068) *Gut Liver* 2011 Mar;5(1):22-8. [doi: 10.5009/gnl.2011.5.1.22. PMID: 21461068]

6. Cui S, Hu Y. [Multistrain probiotic preparation significantly reduces symptoms of irritable bowel syndrome in a double-blind placebo-controlled study](https://www.ncbi.nlm.nih.gov/pmc/articles/PMC3403550/). *Int J Clin Exp Med* 2012; 5(3): 238–244.

7. Dapoigny M, Piche T, Ducrotte P, Lunaud B, Cardot JM, Bernalier-Donadille A. Efficacy and safety profile of LCR35 complete freeze-dried culture in irritable bowel syndrome: a randomized, double-blind study. *World J Gastroenterol* 2012 May 7;18(17):2067-75. [doi: 10.3748/wjg.v18.i17.2067]

8. Drouault-Holowacz S, Bieuvelet S, Burckel A, Cazaubiel M, Dray X, Marteau P. A double blind randomized controlled trial of a probiotic combination in 100 patients with irritable bowel syndrome. *Gastroenterol Clin Biol* 2008 Feb;32(2):147-52.

9. [Enck P, Zimmermann K, Menke G, Müller-Lissner S, Martens U, Klosterhalfen S.](http://www.ncbi.nlm.nih.gov/pubmed/18565142?ordinalpos=2&itool=EntrezSystem2.PEntrez.Pubmed.Pubmed_ResultsPanel.Pubmed_RVDocSum) A mixture of *Escherichia coli* (DSM 17252) and *Enterococcus faecalis* (DSM 16440) for treatment of the irritable bowel syndrome - A randomized controlled trial with primary care physicians. *Neurogastroenterol Motil* 2008;20(10):1103-9. [doi: 10.1111/j.1365-2982.2008.01156.x]

10. Enck P, Zimmermann K, Menke G, Klosterhalfen S. Randomized controlled treatment trial of irritable bowel syndrome with a probiotic *E. coli* preparation (DSM17252) compared to placebo. *Z Gastroen*terol 2009; 2009 Feb;47(2):209-14. [doi: 10.1055/s-2008-1027702]

11. [Fanigliulo L, Comparato G, Aragona G, et al.](http://www.ncbi.nlm.nih.gov/pubmed/17172187?ordinalpos=5&itool=EntrezSystem2.PEntrez.Pubmed.Pubmed_ResultsPanel.Pubmed_RVDocSum) Role of gut microflora and probiotic effects in the irritable bowel syndrome. *Acta Biomed* 2006 Aug;77(2):85-9.

12. Francavilla R, Piccolo M, Francavilla A, et al. Clinical and Microbiological Effect of a Multispecies Probiotic Supplementation in Celiac Patients With Persistent IBS-type Symptoms: A Randomized, Double-Blind, Placebo-controlled, Multicenter Trial. *J Clin Gastroenterol* 2018 Apr 23. [doi: 10.1097/MCG.0000000000001023]

13. Gade J, Thorn P. Paraghurt for patients with irritable bowel syndrome. A controlled clinical investigation from general practice. *Scand J Prim Health Care* 1989 Mar;7(1):23-6.

14. Giannetti E, Maglione M, Alessandrella A, et al. [A Mixture of 3 Bifidobacteria Decreases Abdominal Pain and Improves the Quality of Life in Children With Irritable Bowel Syndrome: A Multicenter, Randomized, Double-Blind, Placebo-Controlled, Crossover Trial.](https://www.ncbi.nlm.nih.gov/pubmed/27306945) *J Clin Gastroenterol* 2017 Jan;51(1):e5-e10.

15. Guglielmetti S, Mora D, Gschwender M, Popp K. Randomised clinical trial: *Bifidobacterium bifidum* MIMBb75 significantly alleviates irritable bowel syndrome and improves quality of life – a double-blind, placebo-controlled study. *Aliment Pharmacol Ther* 2011; 33: 1123–32.

16. Gupta AK, Maity C. Efficacy and safety of *Bacillus coagulans* LBSC in irritable bowel syndrome. *Medicine* 2021.100:3(e23641).

17. Hod K, Sperber AD, Ron Y, et al. [A double-blind, placebo-controlled study to assess the effect of a probiotic mixture on symptoms and inflammatory markers in women with diarrhea-predominant IBS.](https://www.ncbi.nlm.nih.gov/pubmed/28271623) *Neurogastroenterol Motil* 2017 Jul;29(7). [doi: 10.1111/nmo.13037]

18. Hong KS, Kang HW, Im JP, et al. Effect of probiotics on symptoms in Korean adults with irritable bowel syndrome. *Gut Liver* 2009; 3: 101–7.

19. Ishaque SM, Khosruzzaman SM, Ahmed DS, Sah MP. [A randomized placebo-controlled clinical trial of a multi-strain probiotic formulation (Bio-Kult®) in the management of diarrhea-predominant irritable bowel syndrome.](https://www.ncbi.nlm.nih.gov/pubmed/29801486) *BMC Gastroenterol* 2018 May 25;18(1):71. [doi: 10.1186/s12876-018-0788-9]

20. Jafari E, Vahedi H, Merat S, Momtahen S, Riahi A. Therapeutic effects, tolerability and safety of a multi-strain probiotic in Iranian adults with irritable bowel syndrome and bloating. *Arch Iran Med* 2014 Jul;17(7):466-70. [doi: 0141707/AIM.003]

21. [Khodadoostan M](https://www.ncbi.nlm.nih.gov/pubmed/?term=Khodadoostan%20M%5BAuthor%5D&cauthor=true&cauthor_uid=30050882), [Shavakhi A](https://www.ncbi.nlm.nih.gov/pubmed/?term=Shavakhi%20A%5BAuthor%5D&cauthor=true&cauthor_uid=30050882), [Sherafat Z](https://www.ncbi.nlm.nih.gov/pubmed/?term=Sherafat%20Z%5BAuthor%5D&cauthor=true&cauthor_uid=30050882), [Shavakhi A](https://www.ncbi.nlm.nih.gov/pubmed/?term=Shavakhi%20A%5BAuthor%5D&cauthor=true&cauthor_uid=30050882). Effect of Probiotic Administration Immediately and 1 Month after Colonoscopy in Diarrhea-predominant Irritable Bowel Syndrome Patients. [*Adv Biomed Res*](https://www.ncbi.nlm.nih.gov/pubmed/?term=Khodadoostan+and+IBS) 2018;7:94. [doi: 10.4103/abr.abr_216_17]

22. Kim YG, Moon JT, Lee KM, Chon NR, Park H. [The effects of probiotics on symptoms of irritable bowel syndrome]. *Korean J Gastroenterol.* 2006;47(6):413-419.

23. Lewis ED, Antony JM, Crowley DC, et al. Efficacy of *Lactobacillus paracasei* HA-196 and *Bifidobacterium longum* R0165 in alleviating symptoms of irritable bowel syndrome(IBS): A randomized, placebo-controlled study. Nutrients 2020;12:1159. [doi: 10.3390/nu12041159].

24. Lorenzo-Zúñiga V, Llop E, Suárez C, et al. [I.31, a new combination of probiotics, improves irritable bowel syndrome-related quality of life](https://www.ncbi.nlm.nih.gov/pmc/articles/PMC4093724/). *World J Gastroenterol* 2014; 20(26): 8709–8716. [doi 10.3748/wjg.v20.i26.870]

25. Ludidi S, Jonkers DM, Koning CJ, et al. [Randomized clinical trial on the effect of a multispecies probiotic on visceroperception in hypersensitive IBS patients.](https://www.ncbi.nlm.nih.gov/pubmed/24588932) *Neurogastroenterol Motil* 2014 May;26(5):705-14. [doi: 10.1111/nmo.12320]

26. Lyra A, Hillilä M, Huttunen T, et al. Irritable bowel syndrome symptom severity improves equally with probiotic and placebo. *World J Gastroenterol* 2016 Dec 28;22(48):10631-10642.
[doi: 10.3748/wjg.v22.i48.10631]

27. Martoni CJ, Srivastava S, Leyer GJ. *Lactobacillus acidophilus* DDS-1 and *Bifidobacterium lactis* UAB1a-12 improve abdominal pain severity and symptomology in irritable bowel syndrome: Randomized controlled trial. *Nutrients* 2020;12:363. [doi: 10.3390/nu12020363].

28.[Mezzasalma V](https://www.ncbi.nlm.nih.gov/pubmed/?term=Mezzasalma%20V%5BAuthor%5D&cauthor=true&cauthor_uid=27595104), [Manfrini E](https://www.ncbi.nlm.nih.gov/pubmed/?term=Manfrini%20E%5BAuthor%5D&cauthor=true&cauthor_uid=27595104), [Ferri E](https://www.ncbi.nlm.nih.gov/pubmed/?term=Ferri%20E%5BAuthor%5D&cauthor=true&cauthor_uid=27595104), et al. A Randomized, Double-Blind, Placebo-Controlled Trial: The Efficacy of Multispecies Probiotic Supplementation in Alleviating Symptoms of Irritable Bowel Syndrome Associated with Constipation. [*Biomed Res Intl*](https://www.ncbi.nlm.nih.gov/pubmed/?term=Mezzaslana+V+and+IBS) 2016;2016:4740907 [doi: 10.1155/2016/4740907]

29. Murakami K, Habukawa C, Nobuta Y, Moriguchi N, Takemura T. The effect of *Lactobacillus brevis* KB290 against irritable bowel syndrome: a placebo-controlled double-blind crossover trial. *Biopsychosoc Med* 2012; 6: 16. [doi: 10.1186/1751-0759-6-16]

30. Niv E, Naftali T, Hallak R, Vaisman N. The efficacy of *Lactobacillus reuteri* ATCC 55730 in the treatment of patients with irritable bowel syndrome--a double blind, placebo-controlled, randomized study. *Clin Nutr* Dec 2005;24(6):925-931.

31. Oh JH, Jang YS, Kang D, et al. Efficacy and safety of new *Lactobacilli* probiotics for unconstipated irritable bowel syndrome: A randomized, double-blind, placebo-controlled trial. *Nutrients* 2019; 11:2887. [doi: 10.3390/nu11122887].

32. O'Mahony L, McCarthy J, Kelly P, et al. Lactobacillus and bifidobacterium in irritable bowel syndrome: symptom responses and relationship to cytokine profiles. *Gastroenterology* 2005;128(3):541-551.

33. [Preston K](https://www.ncbi.nlm.nih.gov/pubmed/?term=Preston%20K%5BAuthor%5D&cauthor=true&cauthor_uid=29888656), [Krumian R](https://www.ncbi.nlm.nih.gov/pubmed/?term=Krumian%20R%5BAuthor%5D&cauthor=true&cauthor_uid=29888656), [Hattner J](https://www.ncbi.nlm.nih.gov/pubmed/?term=Hattner%20J%5BAuthor%5D&cauthor=true&cauthor_uid=29888656), et al. *Lactobacillus acidophilus* CL1285, *Lactobacillus casei* LBC80R and *Lactobacillus* *rhamnosus* CLR2 improve quality-of-life and IBS symptoms: a double-blind, randomised, placebo-controlled study. [*Benef Microbes*](https://www.ncbi.nlm.nih.gov/pubmed/?term=Preston+K+and+IBS) 2018 Sep 18;9(5):697-706. [doi: 10.3920/BM2017.0105]

34. Ringel Y, Ringel-Kulka T, Maier D, et al. [Clinical trial: Probiotic Bacteria *Lactobacillus acidophilus* NCFM and *Bifidobacterium lacti*s Bi-07 Versus Placebo for the Symptoms of Bloating in Patients with Functional Bowel Disorders - a Double-Blind Study](https://www.ncbi.nlm.nih.gov/pmc/articles/PMC4372813/). *J Clin Gastroenterol* 2011; 45(6): 518–525. [doi: 10.1097/MCG.0b013e31820ca4d6]

35. Roberts LM, McCahon D, Holder R, Wilson S, Hobbs FDR. A randomised controlled trial of a probiotic ‘functional food’ in the management of irritable bowel syndrome. BMC Gastroenterol. 2013; 13: 45. Published online 2013 Mar 7. [doi: 10.1186/1471-230X-13-45]

36. Sadrin S, Sennoune S, Gout B, et al. A 2-strain mixture of *Lactobacillus* *acidophilus* in the treatment of irritable bowel syndrome: A placebo-controlled randomized clinical trial. *Dig Liv Dis* 2020;52:534-540. [doi: 10.1016/j.gld.2019.12.009]

37. Saggioro A. Probiotics in the treatment of irritable bowel syndrome. *J Clin Gastroenterol*  2004;38(6 Suppl):S104-106.

38. Shavakhi A, Tabesh E, Yaghoutkar A, et al. The effects of multi-strain probiotic compound on bismuth-containing quadruple therapy for *Helicobacter pylori* infection: a randomized placebo-controlled triple-blind study *Helicobacter* 2013 Aug;18(4):280-4. [doi: 10.1111/hel.12047]

39. [Shin SP](https://www.ncbi.nlm.nih.gov/pubmed/?term=Shin%20SP%5BAuthor%5D&cauthor=true&cauthor_uid=29610559), [Choi YM](https://www.ncbi.nlm.nih.gov/pubmed/?term=Choi%20YM%5BAuthor%5D&cauthor=true&cauthor_uid=29610559), [Kim WH](https://www.ncbi.nlm.nih.gov/pubmed/?term=Kim%20WH%5BAuthor%5D&cauthor=true&cauthor_uid=29610559), et al. A double blind, placebo-controlled, randomized clinical trial that breast milk derived-*Lactobacillus gasseri* BNR17 mitigated diarrhea-dominant irritable bowel syndrome. [*J Clin Biochem Nutr*](https://www.ncbi.nlm.nih.gov/pubmed/?term=Shin+SP+and+IBS) 2018;62(2):179-186. [doi: 10.3164/jcbn.17-73]

40. Sinn DH, Song JH, Kim HJ, et al. Therapeutic effect of *Lactobacillus acidophilus*-SDC 2012, 2013 in patients with irritable bowel syndrome. *Dig Dis Sci* 2008; 53(10):2714-8. [doi 10.1007/s10620-00700196-4]

41. [Sisson G](https://www.ncbi.nlm.nih.gov/pubmed/?term=Sisson%20G%5BAuthor%5D&cauthor=true&cauthor_uid=24815298), [Ayis S](https://www.ncbi.nlm.nih.gov/pubmed/?term=Ayis%20S%5BAuthor%5D&cauthor=true&cauthor_uid=24815298), [Sherwood RA](https://www.ncbi.nlm.nih.gov/pubmed/?term=Sherwood%20RA%5BAuthor%5D&cauthor=true&cauthor_uid=24815298), [Bjarnason I](https://www.ncbi.nlm.nih.gov/pubmed/?term=Bjarnason%20I%5BAuthor%5D&cauthor=true&cauthor_uid=24815298). Randomised clinical trial: A liquid multi-strain probiotic vs. placebo in the irritable bowel syndrome--a 12 week double-blind study. [*Aliment Pharmacol Ther*](https://www.ncbi.nlm.nih.gov/pubmed/?term=Sisson++and+IBS) 2014 Jul;40(1):51-62. [doi: 10.1111/apt.12787]

42. Skrzydio-Radomanska B, Prozorow-Krol B, Cichoz-Lack H, et al. The effectiveness of synbiotic preparation containing *Lactobacillus* and *Bifidobacterium* probiotic strains and short chain fructooligosaccharides in patients with diarrhea predominant irritable bowel syndrome-A randomized double-blind, placebo-controlled study. *Nutrients* 2020;12:1999. [doi: 10.3390/nu12071999].

43. Sun YY, Li M, Li YY, et al. [The effect of *Clostridium butyricum* on symptoms and fecal microbiota in diarrhea-dominant irritable bowel syndrome: a randomized, double-blind, placebo-controlled trial.](https://www.ncbi.nlm.nih.gov/pubmed/29445178) *Sci Rep* 2018;14;8(1):2964. [doi: 10.1038/s41598-018-21241-z]

44. Thijssen AY, Clemens CH, Vankerckhoven V, Goossens H, Jonkers DM, Masclee AA. [Efficacy of *Lactobacillus casei* Shirota for patients with irritable bowel syndrome.](https://www.ncbi.nlm.nih.gov/pubmed/26469356) *Eur J Gastroenterol Hepatol* 2016;28(1):8-14. [doi: 10.1097/MEG.0000000000000484]

45. Williams EA, Stimpson J, Wang D, et al. Clinical trial: a multistrain probiotic preparation significantly reduces symptoms of irritable bowel syndrome in a double-blind placebo-controlled study. *Aliment Pharmacol Ther* 2009; 29: 97–103.

46. Zeng J, Li YQ, Zuo XL, Zhen YB, Yang J, Liu CH. Clinical trial: effect of active lactic acid bacteria on mucosal barrier function in patients with diarrhea-predominant irritable bowel syndrome. *Aliment Pharmacol Ther* 2008;28(8):994-1002. [doi: 10.1111/j.1365-2036.2008.03818.x.Jul 30]

**Supplementary Table 2. Study population and intervention characteristics in 42 randomized controlled trials in IBS patients treated with either probiotics or controls.**

| **Probiotic** | **Study population  [# enrolled, type of subjects, IBS sub-types, country)** | **Attrition (%)** | **Daily dose (cfu/d)** | **Formulation** | **Duration of treatment (weeks)** | **Duration follow-up (weeks)** | **Reference^a^** |
| --- | --- | --- | --- | --- | --- | --- | --- |
| *Bacillus coagulans* MTCC5260 | N=154 children (4-12 yrs) 43% female Rome III IBS-D (41%)/IBS-C (52%)/IBS-M (7%) India | 8 | 2 x 10^9^ | tablets | 8 | 2 | Sudha 2018^18^ |
| *Bacillus coagulans* MTCC5260 | N=136 adults (18-60 yrs) 28% female Rome III IBS types nr India | 29 | 2 x 10^9^ | capsules | 8 | 2 | Madempudi 2019^19^ |
| *Bacillus coagulans* MTCC5856 | N=52  adults (18-75 yrs) 65% female Rome III IBS types nr Italy | 0 | 2 x 10^9^ | tablet | 4 | 0 | Urgesi 2014^20^ |
| *Bacillus coagulans* MTCC5856 + FOS | N=85  adults (range nr) 78% female Rome III IBS-D (32%)/IBS-C (12%)/IBS-M (50%) Iran | 34 | 4.5 x 10^8^ | tablets | 12 | 36 | Rogha 2014^21^ |

| **Probiotic** | **Study population  [# enrolled, type of subjects, IBS subtypes, country)** | **Attrition** (%) | **Daily dose (cfu/d)** | **Formulation** | **Duration of treatment (weeks)** | **Duration follow-up (weeks)** | **Reference** |
| --- | --- | --- | --- | --- | --- | --- | --- |
| *Bacillus coagulans* MTCC5856 | N=36 adults (18-55 yrs old) 53% female Rome III IBS-D (100%) India | 14 | 2 x 10^9^ | tablet | 12 | 2 | Majeed 2016^22^ |
| *Bifidobacterium infantis* 35624 | N=53 adults (18-75 yrs) 64% female Rome II IBS-D (28%)/IBS-C (26%)/IBS-M (45%) Ireland | 7.5 | 1 x 10^10^ | drink | 8 | 4 | O’Mahony 2005^23^ |
| *Bifidobacterium infantis* 35624 | N=362 women adult (18-65 yrs) 100% female Rome II IBS-D (55%)/IBS-C (21%)/IBS-M (24%) U.K. | 18 | 1 x 10^6^ | capsule | 4 | 2 | Whorwell 2006^24^ low dose |
| *Bifidobacterium infantis* 35624 |  | 20 | 1 x 10^8^ | capsule | 4 | 2 | Whorwell 2006^24^ medium dose |
| *Bifidobacterium infantis* 35624 |  | 21 | 1 x 10^10^ | capsule | 4 | 2 | Whorwell 2006^24^ high dose |
| *Bifidobacterium animalis*  DN-173 010 | N=274 adults (18-65 yrs) 74% female Rome II IBS-C (100%) FRANCE | 3 | 2.5 x 10^10^ | yogurt | 6 | none | Guyonnet 2007^25^ |
| *Bifidobacterium animalis*  DN-173 010 | N=41 women adults (20-69 yrs) 100% female Rome III IBS-C (100%) U.K. | 16 | 2 x 10^10^ | yogurt | 4 | 5 | Agrawal 2009^26^ |

| **Probiotic** | **Study population  [# enrolled, type of subjects, IBS subtypes, country)** | **Attrition** (%) | **Daily dose (cfu/d)** | **Formulation** | **Duration of treatment (weeks)** | **Duration follow-up (weeks)** | **Reference** |
| --- | --- | --- | --- | --- | --- | --- | --- |
| *Escherichia coli* Nissle 1917 | N=120  adults (18-65 yrs) 77% female Rome II IBS-D (34%)/IBS-C (22%)/IBS-M (44%) Germany | 17.5 | 5 x 10^10^ | capsule | 12 | none | Kruis 2012^27^ |
| *Escherichia coli* Nissle 1917 | N=150  adults (20-50 yrs) 51% female Rome II  IBS-D (35%)/IBS-C (40%)/IBS-M (25%) Iran | 30 | nr | capsule | 6 | none | Faghihi 2015^28^ |
| *Lactobacillus plantarum* 299v DSN9843 | N=60  adults (>18 yrs) 64% female Rome II IBS types nr Sweden | 13 | 2 x 10^10^ | drink | 4 | 52 | Nobaek 2000^29^ |
| *Lactobacillus plantarum* 299v DSN9843 | N=40 adults (27-63 yrs) 75% female Manning criteria IBS-D (2%), IBS-C (52%), IBS-M (45%) Poland | 0 | 2 x 10^10^ | drink | 4 | none | Niedezielin 2001^30^ |
| *Lactobacillus plantarum* 299v DSN9843 | N=66 adults (range nr) 73% female Rome II IBS types nr Sweden | 12 | 2 x 10^10^ | drink | 6 | none | Simren 2006^31^ |
| *Lactobacillus plantarum* 299v DSN9843 | N=214 adults (18-70 yrs) 29% female Rome III IBS-D (64%)/others nr India | 5 | 1 x 10^10^ | capsule | 4 | 3 | Ducrotte 2012^32^ |

| **Probiotic** | **Study population** | **Attrition (%)** | **Daily dose (cfu/d)** | **Formulation** | **Duration of treatment (weeks)** | **Duration follow-up (weeks)** | **Reference** |
| --- | --- | --- | --- | --- | --- | --- | --- |
| *L. plantarum 299v DSN9843* | N=81 adults (>18 yrs) 97% female Rome II IBS-D (50%)/IBS-C (50%) South Africa | 20 | 1 x 10^10^ | capsule | 8 | 2 | Stevenson 2014^33^ |
| *Lactobacillus rhamnosus GG* | N=64  children (6-20 yrs)  80% female Rome II IBS types nr U.S.A | 22% | 2 x 10^10^ | capsule | 6 | none | Bausserman 2005^34^ |
| *Lactobacillus rhamnosus GG* | N=37  children (6-16 yrs)  Rome II % female nr IBS types nr Poland | 0% | 6 x 10^9^ | capsule | 4 | none | Gawronska 2007^35^ |
| *Lactobacillus rhamnosus GG* | N=83 IBS sub-group children (5-14 yrs) 37% female Rome II IBS types nr Italy | 3.5% | 6 x 10^8^ | capsule | 8 | 8 wks | Francavilla 2010^36^ |
| *Lactobacillus rhamnosus GG* | N=123  adults (18-74 yrs)  73% female Rome III  IBS-D (41%)/IBS-C (15%)/IBS-M (38%) Denmark | 12% | 1.2 x 10^10^ | capsule | 6 | none | Pedersen 2014^37^ |

| **Probiotic** | **Study population  [# enrolled, type of subjects, IBS subtypes, country)** | **Attiriton (%)** | **Daily dose (cfu/d)** | **Formulation** | **Duration of treatment (weeks)** | **Duration follow-up (weeks)** | **Reference** |
| --- | --- | --- | --- | --- | --- | --- | --- |
| *Lactobacillus rhamnosus GG* | N=60  children (4-18 yrs)  48% female Rome III  IBS-D (17%)/IBS-C (23%)/IBS-M (60%) Iran | 13 | 2 x 10^10^ | capsule | 4 | none | Kianifar 2015^38^ |
| *S. boulardii* CNCM I-745 | N=34  adults (>18 yrs) 41% female Rome criteria nr IBS types nr France | 0 | 9 x 10^9^ | capsule | 4 | none | Maupas 1983^39^ |
| *S. boulardii* CNCM I-745 | N=55 adults 69% female IBS-D (44%)/IBS-C (16%)/IBS-A (29%) France | 0 | 1 x 10^10^ | capsule | 4 | 8 | Bennani 1990^40^ |
| *S. boulardii* CNCM I-745 | N=90  adults (20-65 yrs) 50% female Rome II IBS-D (72%)/IBS-M (28%) Korea | 26 | 8 x 10^11^ | capsule | 4 | none | Choi 2011^41^ |
| *S. boulardii* CNCM I-745 | N=70  adults (18-50 yrs) 8% female Rome II IBS-D (100%) Bangladesh | 0 | 1 x 10^10^ | sachets | 4 | 4 | Kabir 2011^42^ |
| *S. boulardii* CNCM I-745 | N=72  adults (18-60 yrs)  26% female Rome III IBS-D (100%) Pakistan | 11 | 1.5 x 10^10^ | capsule | 6 | none | Abbas 2014^43^ |

| **Probiotic** | **Study population** | **Attrition (%)** | **Daily dose (cfu/d)** | **Formulation** | **Duration of treatment (weeks)** | **Duration follow-up (weeks)** | **Reference** |
| --- | --- | --- | --- | --- | --- | --- | --- |
| *S. cerevisiae* I-3856 | N=200 adults (18-75 yrs)  86% female Rome III IBS-D (28%)/IBS-C (47%)/IBS-M (25%) France | 10.5 | 4 x 10^9^ | capsule | 8 | 3 | Pineton de Chambrun 2015^44^ |
| *S. cerevisiae* I-3856 | N=379  adults (18-75 yrs)  84% female Rome III IBS-D (21%),IBS-C (47%), IBS-M (32%) France | 4 | 8 x 10^9^ | capsule | 12 | none | Spiller 2016 ^45^ |
| *S. cerevisiae* I-3856 | N=100 adults (>18 yrs) 34% female Rome III IBS-D (65%)/IBS-C (24%)/IBS-M (11%) India | 8 | 4 x 109 | capsule | 8 | 2 | Gayathri 2020^46^ |

| **Probiotic** | **Study population  [# enrolled, type of subjects, IBS subtypes, country)** | **Attrition** (%) | **Daily dose (cfu/d)** | **Formulation** | **Duration of treatment (weeks)** | **Duration follow-up (weeks)** | **Reference** |
| --- | --- | --- | --- | --- | --- | --- | --- |
| “Cultura” *L. paracasei 19, L. acido La5, Bifi lactis Bb12* | N=118  adults (18-70 yrs) 70% female  Rome II IBS-D (35%)/IBS-C (15%)/IBS-M (50%) Sweden | 9.5 | 1 x 10^10^ | milk | 8 | 8 | Simren 2010^47^ |
| “Cultura” *L. paracasei 19, L. acido La5, Bifi lactis Bb12* | N=64  adults (18-70 yrs) 75% female Rome II IBS types nr Sweden & Denmark | 18.8 | 2.5 x 10^10^ | milk | 8 | 8 | Sondergaard 2011^48^ |
| “Cultura” *L. paracasei 19, L. acido La5, Bifi lactis Bb12* | N=131 adults (18-50 yrs)  74% female Rome III IBS-D (40%)/IBS-C (19%)/IBS-M (38%) Denmark | 24 | 5.2 x 10^10^ | capsule | 244 | 24 | Begtrup 2013^49^ |
| 4 strains: *L. rhamnosus* GG, *L. rhamnosus* LC705, *Bifido. breve* Bb99, *P.* freudenreichii ssp. shermanii JS | N=103  adults (20-65 yrs) 77% female Rome II  IBS-D (48%)/IBS-C (23%)/IBS-M (29%) Finland | 21.3 | 8-9 x 10^9^ | capsule | 24 | none | Kajander 2005^50^ |
| 4 strains: *L. rhamnosus* GG, *L. rhamnosus* LC705, *Bifido. breve* Bb99, *P.* freudenreichii ssp. shermanii JS | N=86  adults (20-65 yrs)  95% female Rome II  IBS-D (45%)/IBS-C (30%)/IBS-M (24%)  Finland | 17 | 4.8 x 10^10^ | milk | 20 | 3 | Kajander 2008^51^ |

| **Probiotic** | **Study population** | **Attrition (%)** | **Daily dose (cfu/d)** | **Formulation** | **Duration of treatment (weeks)** | **Duration follow-up (weeks)** | **Reference** |
| --- | --- | --- | --- | --- | --- | --- | --- |
| 6 strains “LacClean Mix”: *L. acidophilus* 11906BP, *L. rhamnosus* 12202BP, *Bifido. bifidum* 12199BP, *Bifido. lacti*s 11904 BP, *Bifido. longum* 12200 BP, *Strept. thermophius* 11870BP | N=49  adults (19-75 yrs) 65% female Rome III IBS-D (53%)/IBS-C (41%)/IBS-M (6%) Korea | 0 | 1 x 10^10^ | capsule | 4 | none | Yoon 2014^52^ |
| 6 strains “LacClean Mix”: *L. acidophilus* 11906BP, *L. rhamnosus* 12202BP, *Bifido. bifidum* 12199BP, *Bifido. lacti*s 11904 BP, *Bifido. longum* 12200 BP, *Strept. thermophius* 11870BP | N=95  adults (19-75 yrs) 53% female  Rome III  IBS-D (48%)/IBS-C (18%)/IBS-M (21%)/unknw (12%) Korea | 15 | 1 x 10^10^ | capsule | 4 | none | Yoon 2015^53^ |
| 7 strains: “DuoLac”: *Bifido. brevis* 11858BP, *Bifido. lactis* 11903BP, *Bifido. longum* 11860BP, *L. acidophilus* 11906BP,*L. rhamnosus* 11868BP, *L. plantarum* 11867BP, *Strept thermophilus* 11870BP | N=50  adults (18-65 yrs) 48% female Rome III  IBS-D (100%) Korea | 6 | 2 x 10^10^ | capsule | 8 | 2 | Ki Cha 2012^54^ |
| Probiotic mix only vs placebo: 7 strains: “DuoLac”: *Bifido. brevis* 11858BP, *Bifido. lactis* 11903BP, *Bifido. longum* 11860BP, *L. acidophilus* 11906BP,*L. rhamnosus* 11868BP, *L. plantarum* 11867BP, *Strept thermophilus* 11870BP | N=26  adults (18-75 yrs) 36% female Rome III IBS-D (100%) Korea | 15 | 1 x 10^9^ | capsule | 8 | 2 | Ko 2013^55^ |
| Probiotic mix only vs placebo: 7 strains: “DuoLac”: *Bifido. brevis* 11858BP, *Bifido. lactis* 11903BP, *Bifido. longum* 11860BP, *L. acidophilus* 11906BP,*L. rhamnosus* 11868BP, *L. plantarum* 11867BP, *Strept thermophilus* 11870BP | N=27  adults (18-75 yrs) 36% female Rome III IBS-D (100%) Korea | 4 | 1 x 10^9^ | capsule | 8 | 2 | Ko 2013^55^ |

| **Probiotic** | **Study population** | **Attrition (%)** | **Daily dose (cfu/d)** | **Formulation** | **Duration of treatment (weeks)** | **Duration follow-up (weeks)** | **Reference** |
| --- | --- | --- | --- | --- | --- | --- | --- |
| 8-strain mix | N=25  adults (18-75 yrs)  72% female Rome II  IBS-D (100%)  U.S.A. | 4 | 9 x 10^11^ | powder | 8 | none | Kim 2003^56^ |
| 8-strain mix | N=48  adults (18-75 yrs) 94% female  Rome II IBS-D (42%)/IBS-C (33%)/IBS-M (25%) U.S.A. | 0 | 8 x 10^11^ | yogurt | 4 | none | Kim 2005^57^ |
| 8-strain mix | N=59 children (4-18 yrs) 47% female Rome II IBS-D (37%)/IBS-C (34%)/IBS-M (39%) India and Italy | 12 | 4-8 x 10^11^ | sachet | 6 | 2 | Guandalini 2010^58^ |
| 8-strain mix | N=104  adults (18-65 yrs) 67% female Rome III IBS-D (66%)/IBS-C (0%)/IBS-M (23%)/IBS-unknown (11%) U.K.. | 16 | 9 x 10^11^ | sachet | 4 | none | Staudaeher 2017^59^ |

^a^ Reference numbers in main paper

**Abbreviations**: Bifido., *Bifidobacterium*; L., *Lactobacillus*; N, number; nr, not reported; P., Propionibacterium; S., *Saccharomyces*; Strept., *Streptococcus*; wks, weeks; 8-strain mix,: *Bifido. breve* DSM24732, *Bifido. longum* DSM24736, *Bifido. infantis* DSM24737, *L. acidophilus* DSM24735, *L. plantarum* DSM24730, *L. paracasei* DSM24733, *L. delbruckii* subsp. *bulgaricus* DSM24734, *Strept. thermophiles* DSM24731, originally named VSL#3®, now either Visbiome™ or Vivomixx™ using the De Simone formulation; yrs, years.

**Supplementary Table 3. Adverse reactions and safety in 42 randomized controlled trials in IBS patients treated with either probiotics or controls.**

| **Probiotic** | **Adverse effects (AEs) data** | **Frequency AEs in probiotic group** | **Frequency AEs in controls** | **Types of AEs reported per group** | **Serious AEs reported in probiotic** | **Serious AEs reported in controls** | **Reference^a^** |
| --- | --- | --- | --- | --- | --- | --- | --- |
| *B. coagulans* MTCC52606 | yes^b^ | nr | nr | nr | 0 (0%) | 0 (0%) | Sudha 2018^18^ |
| *B. coagulans* MTCC5260 | yes^b^ | 0 | 0 | no | 0 (0%) | 0 (0%) | Madempudi 2019^19^ |
| *B. coagulans* MTCC5856 | no | nr | nr | nr | 0 (0%) | 0 (0%) | Urgesi 2014^20^ |
| *B. coagulans* MTCC5856 | yes | 41% | 26% | yes | 0 (0%) | 0 (0%) | Rogha 2014^21^ |
| *B. coagulans* MTCC5856 | yes | 6% | 0% | yes | 0 (0%) | 0 (0%) | Majeed 2016^22^ |
| *Bifido. infantis* 35624 | yes | nr | nr | no | nr | nr | O’Mahony 2005^23^ |
| *Bifido. infantis* 35624 | yes | nr | nr | no | nr | nr | Whorwell 2006^24^ low dose |
| *Bifido. infantis* 35624 | yes | nr | nr | no | nr | nr | Whorwell 2006^24^ medium dose |
| *Bifido. infantis* 35624 | yes | nr | nr | no | nr | nr | Whorwell 2006^24^ high dose |
| *Bifido. animalis* DN-173 010 | yes | 9.6% | 7.6% | yes | 0 (0%) | 2 (1%) | Guyonnet 2007^25^ |
| *Bifido. animalis* DN-173 010 | no | nr | nr | no | nr | nr | Agrawal 2009^26^ |
| *E. coli* Nissle 1917 | yes | 50% | 45% | yes | 0 (0%) | 1 (2%) | Kruis 2012^27^ |
| *E. coli* Nissle 1917 | no | nr | nr | nr | nr | nr | Faghihi 2015^28^ |
| *L. plantarum* 299v DSN9843 | yes^b^ | nr | nr | nr | nr | nr | Nobaek 2000^29^ |
| *L. plantarum* 299v DSN9843 | yes^b^ | nr | nr | nr | nr | nr | Niedezielin 2001^30^ |
| *L. plantarum* 299v DSN9843 | yes^b^ | nr | nr | nr | nr | nr | Simren 2006^31^ |
| *L. plantarum* 299v DSN9843 | yes | 0.9% | 0% | yes | 0.9% | 0% | Ducrotte 2012^32^ |
| *L. plantarum* 299v DSN9843 | yes | 1.9% | 0% | yes | 0 | 0 | Stevenson 2014^33^ |

| **Probiotic** | **Adverse effects (AEs) data** | **Frequency AEs in probiotic group** | **Frequency AEs in controls** | **Types of AEs reported per group** | **Serious AEs reported in probiotic** | **Serious AEs reported in controls** | **Reference** |
| --- | --- | --- | --- | --- | --- | --- | --- |
| *L. rhamnosus* GG | yes^b^ | nr | nr | nr | nr | nr | Bausserman 2005^34^ |
| *L. rhamnosus* GG | yes^b^ | nr | nr | nr | nr | nr | Gawronska 2007^35^ |
| *L. rhamnosus* GG | yes^b^ | nr | nr | nr | nr | nr | Francavilla 2010^36^ |
| *L. rhamnosus* GG | no | nr | nr | nr | nr | nr | Pedersen 2014^37^ |
| *L. rhamnosus* GG | yes^b^ | nr | nr | nr | nr | nr | Kianifar 2015^38^ |
| *S. boulardii* CNCM I-745 | yes^b^ | nr | nr | nr | nr | nr | Maupas 1983^39^ |
| *S. boulardii* CNCM I-745 | yes | nr | nr | nr | nr | nr | Bennani 1990^40^ |
| *S. boulardii* CNCM I-745 | yes | 0% | 2% | yes | nr | nr | Choi 2011^41^ |
| *S. boulardii* CNCM I-745 | no | nr | nr | nr | nr | nr | Kabir 2011^42^ |
| *S. boulardii* CNCM I-745 | yes | 51% | 43% | no | 0 (0%) | 0 (0%) | Abbas 2014^43^ |
| *S. cerevisiae* CNCM I-3856 | yes | 50% | 50% | no | 0 (0%) | 0 (0%) | Pineton de Chambrun 2015^44^ |
| *S. cerevisiae* CNCM I-3856 | yes | 7% | 3% | yes | 0 (0%) | 0 (0%) | Spiller 2016 ^45^ |
| *S. cerevisiae* CNCM I-3856 | yes | 13% | 6% | yes | 0 (0%) | 0 (0%) | Gayathri 2020^46^ |
| L. paracasei 19, L. acido La5, Bifi lactis Bb12 | no | nr | nr | nr | nr | nr | Simren 2010^47^ |
| L. paracasei 19, L. acido La5, Bifi lactis Bb12 | yes^b^ | nr | nr | nr | nr | nr | Sondergaard 2011^48^ |
| L. paracasei 19, L. acido La5, Bifi lactis Bb12 | yes | 3% | 0% | yes | 0 (0%) | 0 (0%) | Begtrup 2013^49^ |
| 4 strains: *L. rhamnosus* GG, *L. rhamnosus* LC705, *Bifido. breve* Bb99, *P.* freudenreichii ssp. shermanii JS | yes | 7% | 2% | yes | nr | nr | Kajander 2005^50^ |
| 4 strains: *L. rhamnosus* GG, *L. rhamnosus* LC705, *Bifido. breve* Bb99, *P.* freudenreichii ssp. shermanii JS | yes | 23% | 35% | yes | nr | nr | Kajander 2008^51^ |

| **Probiotic** | **Adverse effects (AEs) data** | **Frequency AEs in probiotic group** | **Frequency AEs in controls** | **Types of AEs reported per group** | **Serious AEs reported in probiotic** | **Serious AEs reported in controls** | **Reference** |
| --- | --- | --- | --- | --- | --- | --- | --- |
| 6 strains: *L. acidophilus* 11906BP,  *L. rhamnosus* 12202BP, *Bifido. bifidum* 12199BP, *Bifido. lactis* 11904 BP, *Bifido. longum* 12200 BP, *Strept. thermophilus* 11870BP | yes^b^ | nr | nr | nr | nr | nr | Yoon 2014^52^ |
| 6 strains: *L. acidophilus* 11906BP,  *L. rhamnosus* 12202BP, *Bifido. bifidum* 12199BP, *Bifido. lactis* 11904 BP, *Bifido. longum* 12200 BP, *Strept. thermophilus* 11870BP | no | nr | nr | nr | nr | nr | Yoon 2015^53^ |
| 7 strains: *Bifido. brevis* 11858BP, *Bifido. lactis* 11903BP, *Bifido. longum* 11860BP, *L. acidophilus* 11906BP, *L. rhamnosus* 11868BP, *L. plantarum* 11867BP, *Strept. thermophilus* 11870BP | yes | 0% | 8% | yes | 0 (0%) | 0 (0%) | Ki Cha 2012^54^ |
| Probiotic only vs. placebo: 7 strains: *Bifido. brevis* 11858BP, *Bifido. lactis* 11903BP, *Bifido. longum* 11860BP, *L. acidophilus* 11906BP, *L. rhamnosus* 11868BP, *L. plantarum* 11867BP, *Strept. thermophilus* 11870BP | yes | nr | nr | no | 0 (0%) | 0 (0%) | Ko 2013^55^ |
| Herbal txt + Probiotic mix only vs Herbal only: 7 strains: *Bifido. brevis* 11858BP, *Bifido. lactis* 11903BP, *Bifido. longum* 11860BP, *L. acidophilus* 11906BP, *L. rhamnosus* 11868BP, *L. plantarum* 11867BP, *Strept. thermophilus* 11870BP | yes | nr | nr | no | 0 (0%) | 0 (0%) | Ko 2013^55^ DUO + Herbal vs Herbal only control |
| 8 strain mix | yes^b^ | nr | nr | nr | nr | nr | Kim 2003^56^ |
| 8 strain mix | yes^b^ | nr | nr | nr | nr | nr | Kim 2005^57^ |
| 8 strain mix | yes^b^ | nr | nr | nr | 0 (0%) | 0 (0%) | Guandalini 2010^58^ |
| 8 strain mix | yes | 4% | 8% | yes | 0 (0%) | 0 (0%) | Staudaeher 2017^59^ |

^a^ Reference numbers in main paper

^b^only data provided was text similar to “No adverse effects/event were noted in the study”

**Abbreviations**: AEs, adverse effects; Bifido., *Bifidobacterium*; L., *Lactobacillus;* N, number; nr, not reported; P., Propionibacterium; S., *Saccharomyces*; Strept., *Streptococcus*; wks, weeks; 8-strain mix, *Bifido. breve* DSM24732, *Bifido. longum* DSM24736, *Bifido. infantis* DSM24737, *L. acidophilus* DSM24735, *L. plantarum* DSM24730, *L. paracasei* DSM24733, *L. delbruckii* subsp. *bulgaricus* DSM24734, *Strept. thermophiles* DSM24731, originally named VSL#3®, now either Visbiome™ or Vivomixx™ using the De Simone formulation.


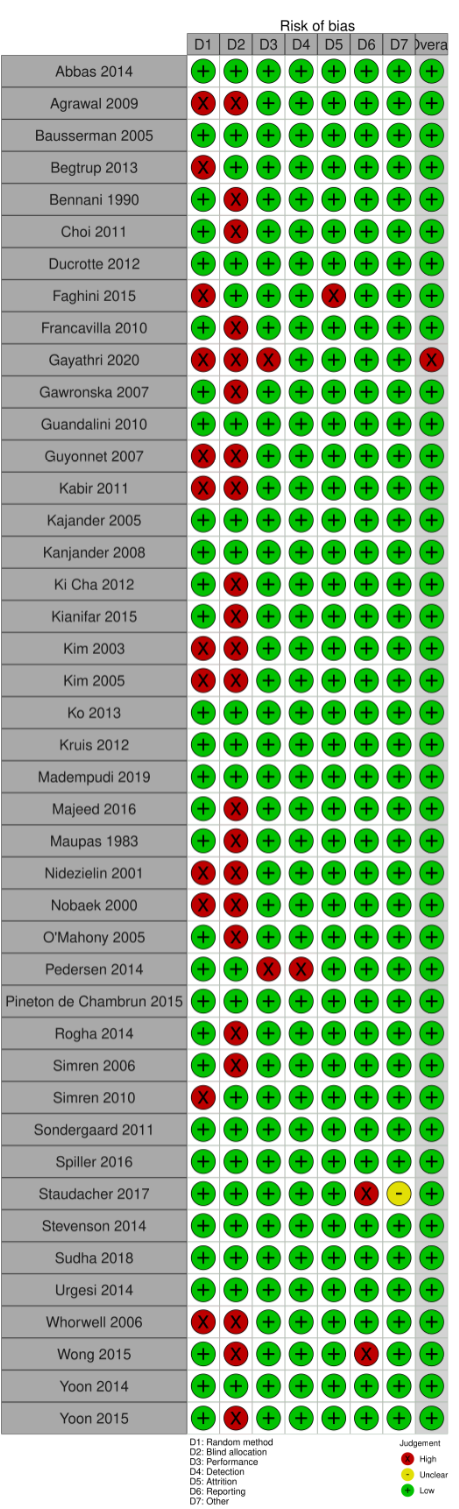


**Supplementary Figure 1. Risk of Bias in included trials of probiotics for treatment of IBS.**

**
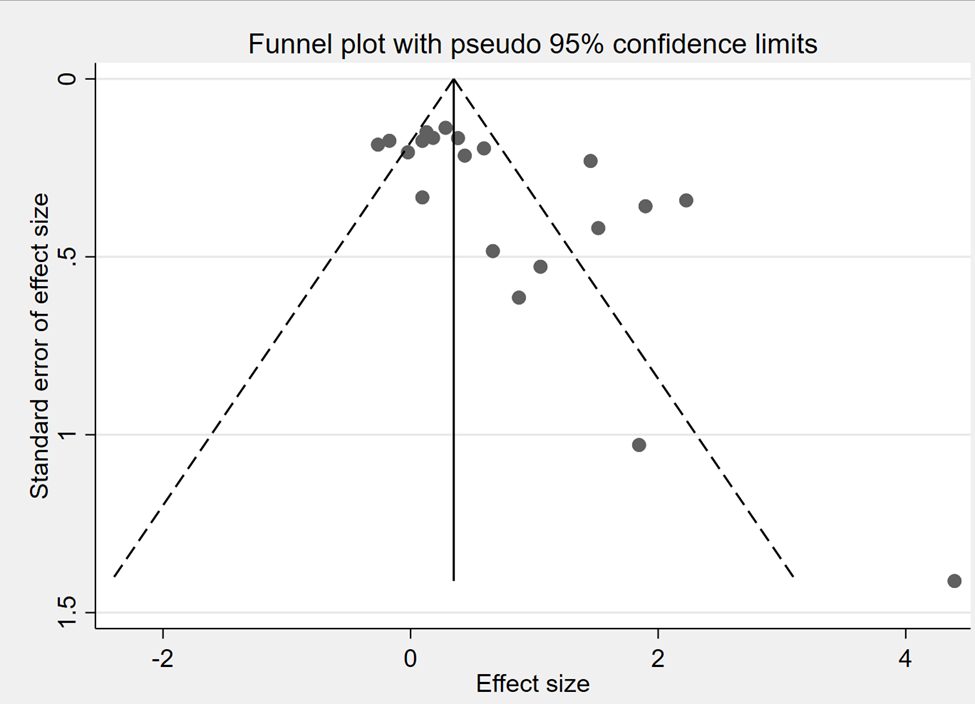
**

**Supplementary Figure 2. Funnel plot for publication bias for IBS probiotic trials with common outcome of “Frequency of no abdominal pain”**

**Supplementary Figure 3. Forest plot of probiotics for global cure “responders” for IBS.**

**Abbreviations**: B inf 35624, *Bifido. infantis* 35624; Lp 299v, *L. plantarum* 299v; *S. boulardii* I-745, *Saccharomyces* *boulardii* CNCM I-745; LpLaBl, *L. paracasei* 19, *L. acido* La5, *Bifido lactis* Bb12; 6 strains: *L. acidophilus* 11906BP, *L. rhamnosus* 12202BP, *Bifido. bifidum* 12199BP, *Bifido. lactis* 11904 BP, *Bifido. longum* 12200 BP, *Strept. thermophilus* 11870BP; VSL#3”: *Bifido. breve* DSM24732, *Bifido. longum* DSM24736, *Bifido. infantis* DSM24737, *L. acidophilus* DSM24735, *L. plantarum* DSM24730, *L. paracasei* DSM24733, *L. delbruckii* subsp. *bulgaricus* DSM24734, *Strept. thermophilus* DSM24731, originally named VSL#3®, now either Visbiome™ or Vivomixx™ using the De Simone formulation.


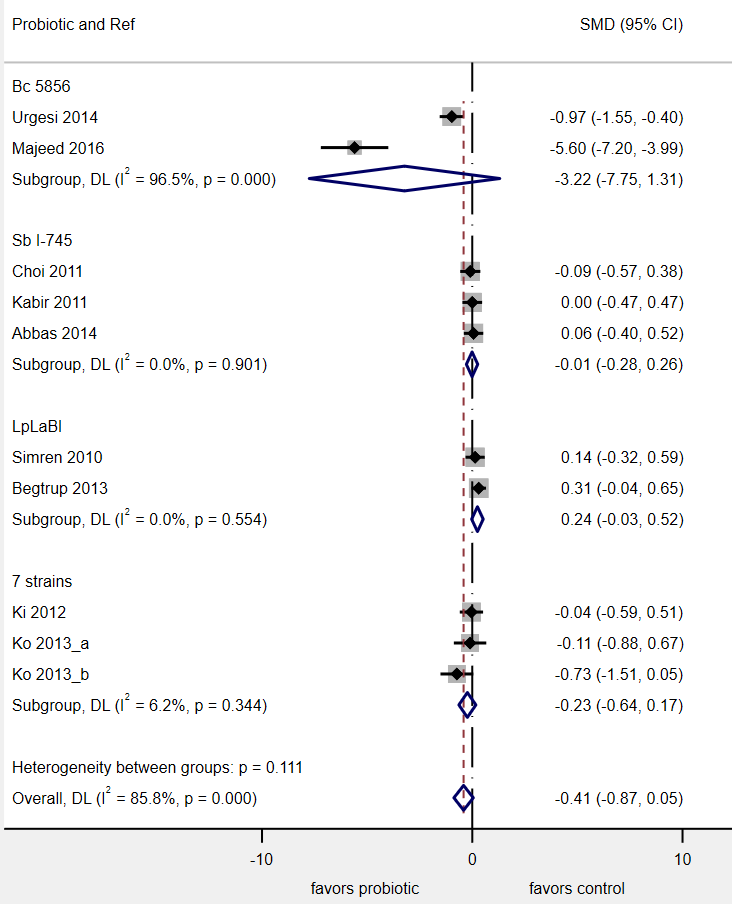


, p<0.001

**Supplementary Figure45. Change in IBS bloating scores by probiotic type.**

**Abbreviations**: **Bc**, *Bacillus coagulans*; **Sb**, *Saccharomyces boulardii*; **LpLaBl**, *L. paracasei 19, L. acido La5, Bifid lactis Bb12;* **7 strains**, *Bif. brevis* 11858BP, *Bif. lactis* 11903BP, *B. longum* 11860BP, *L. acidophilus* 11868BP*, L. rhamnosus* 11868BP*, L. plantarum* 11867BP and *Strept. thermophilus* 11870BP.

**Supplementary Figure 5. Forest plot of randomized controlled trials in patients with IBS-D.**

**Abbreviations**: **Sb**, *Saccharomyces boulardii*; **7 strains**, *Bif. brevis* 11858BP, *Bif. lactis* 11903BP, *B. longum* 11860BP, *L. acidophilus* 11868BP*, L. rhamnosus* 11868BP*, L. plantarum* 11867BP and *Strept. thermophilus* 11870BP.

**
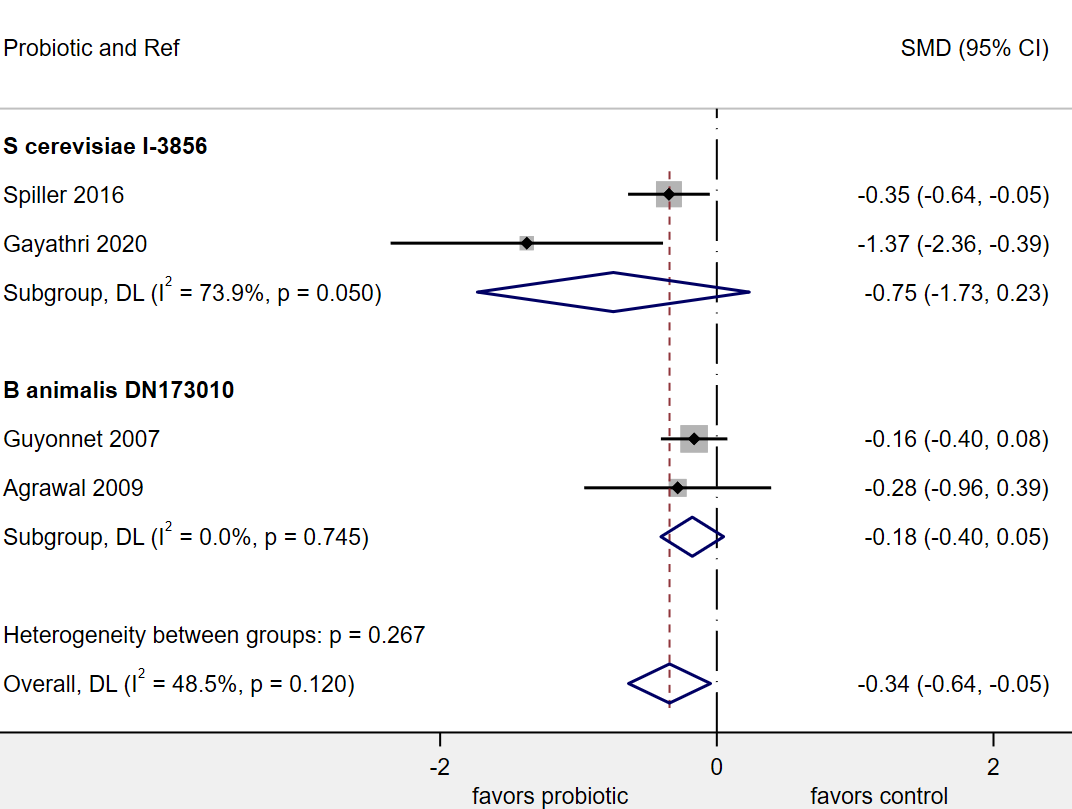
**

**Supplementary Figure 6. Forest plot of randomized controlled trials in patients with IBS-C.**

**Abbreviations**: **S**, *Saccharomyces*; **B**, *Bifidobacterium*.
